# Applying the S-ART Framework to Yoga: Exploring the Self-Regulatory Action of Yoga Practice in Two Culturally Diverse Samples

**DOI:** 10.3389/fpsyg.2021.585300

**Published:** 2021-07-26

**Authors:** Laura Tolbaños-Roche, Praseeda Menon

**Affiliations:** ^1^Department of Clinical Psychology, Psychobiology and Methodology, Section of Psychology, Faculty of Health Sciences, Universidad de La Laguna, San Cristóbal de La Laguna, Spain; ^2^Scientific Research Department, Kaivalyadhama Yoga Institute, Lonavala, India

**Keywords:** yoga, S-ART model, self-regulatory action, well-being, yoga practitioners

## Abstract

Mindfulness practices form the core of numerous therapeutic programs and interventions for stress reduction and the treatment of different health conditions related to stress and life habits. Ways and means to regulate oneself effectively also form the foundation of the path of yoga in the accomplishment of holistic health and well-being. The *self-awareness, self-regulation, and self-transcendence* (S-ART) model can be considered as an overarching neurobiological framework to explain the self-regulatory mechanisms of well-being present in mindfulness-based practices. The current study, by connecting and applying the S-ART framework to the self-regulatory mechanisms in yoga and generating related hypotheses, provides a theory-led explanation of the action of yoga practices, which is sparse in the literature. Testing the S-ART model in yoga in two culturally diverse samples, assessing the model-mapped psychological mechanisms of action, and exploring the influence of perseverance in yoga practice are the original contributions of this study. The study sample comprised 362 yoga practitioners and non-practitioners (197 Indian and 165 Spanish), who completed four tests of psychological variables indicative of the aforementioned three S-ART abilities. These tests were Multidimensional Assessment of Interoceptive Awareness (MAIA), Experiences Questionnaire-Decentering (EQ-D) subscale, Difficulties in Emotion Regulation Scale (DERS), and Relational Compassion Scale (RCS). The results indicated significantly better s*elf-awareness* and *self-regulatory* abilities in yoga practitioners (Indian and Spanish in a combination) than non-practitioners, reflected in higher levels of interoceptive awareness and decentering abilities. Moreover, perseverance in yoga practice acted as a significant predictor of *self-awareness* and *self-regulation* in practitioners. An analysis of each cultural sample revealed some differences. Yoga practice and perseverance in it acted as a significant predictor of interoceptive awareness and decentering in Indian practitioners having more than 1 year of sustained yoga practice, but for the Spanish participants, physical exercise and frequency of yoga practice acted as better predictors of interoceptive awareness and decentering in comparison to yoga practice and perseverance in it. The obtained results suggested that the S-ART model provided preliminary but promising evidence for the self-regulatory mechanisms of action in yoga practice within a culturally diverse sample of yoga practitioners. This study also widens the scope of generating further hypotheses using the S-ART theoretical framework for testing the self-regulatory mechanisms of action in yoga practice.

## Introduction

### Mindfulness and Yoga

Mindfulness practices form the core of numerous therapeutic programs and interventions for stress reduction and the treatment of different health conditions related to stress and life habits. The foundation of these therapeutic programs was based on an important school of thought and practice that emerged in recent years within the western cognitive-behavioral therapeutic approach. This approach itself is rooted in ancient Buddhist practices of meditation, such as *Sattipatthāna* meditation, involving the cultivation of the mindfulness faculty as a means for generating concentration and insight (*vipassanā*, or clear insight). The most significant intervention programs based on this approach, which have also been applied in most of the published research, are the Mindfulness-Based Stress Reduction (MBSR) program (Kabat-Zinn, 1990) and Mindfulness-Based Cognitive Therapy (MBCT) program (Segal et al., 2002).

The popularity of yoga, an Eastern (Indian) mind-body practice, has been growing year by year worldwide. Ways and means to regulate oneself effectively form the foundation of the path of yoga in the accomplishment of holistic health and well-being. However, in many cases, yoga, out of its cultural context, is used only for fitness or, at best, as a body-mind relaxing technique. Over the past two decades, a growing body of research proving yoga's healing benefits has promoted the development of yoga therapy as a clinical treatment for many health conditions (Pascoe and Bauer, 2015; Kumar et al., 2016; Haider et al., 2017; Park and Han, 2017; Thind et al., 2017; Falkenberg et al., 2018). Scientific evidence as well as the effort of many yoga institutions, teachers, and therapists, which have spread genuine yoga teaching for long, have contributed to the universal essence of yoga.

#### Modern and Traditional Views

From the contemporary Western perspective, mindfulness can be defined as “the awareness that emerges through paying attention on purpose, in the present moment, and non-judgmentally to the unfolding of experience moment to moment” (Kabat-Zinn, 2003). Beyond this definition, from the Buddhist traditional perspective, mindfulness is the discriminative awareness for encoding and registering experiences, considerably associated with ethical development as a direct path to “enlightenment” and the cessation of suffering (Chiesa, 2013).

The modern view of yoga considers it as a philosophy-based set of practices and attitudes that focus on the attainment and maintenance of mind-body balance, reducing psycho-physiological activation and facilitating a state of mental calmness as a natural and positive state of mind. The holistic approach of yoga takes into consideration all aspects related to health in an integrative manner: healthy diet, physical activity, breathing techniques, positive thoughts and habits, a health-promoting lifestyle, the regulation of emotions and emotional balance, and a creative cognitive process. The systematic stages of yoga may be thought of as a set of methods to regulate emotions, thoughts, or behavior to promote well-being (Gard et al., 2014).

The traditional or classical approach of yoga (as per Sage *Patanjali's Yogasutra*, the first exclusively written systematization of yoga principles and practices in a highly compact verse form) considers the path of yoga to be a disciplined way of life for the spiritual seeker, a way that leads to the removal of the root cause of human suffering. This root cause is identified as “ignorance” or *avidyā* (*a* = absence of + *vidyā* = the real knowledge worth knowing for spiritual aim; Karambelkar, 1986, p. 177). More precisely, yoga is equated with “the complete cessation of the functional modifications of the *citta*.” Although there is no exact equivalent in English, the c*itta* could be translated as the “mind” for practical understanding (Karambelkar, 1986, p. 5–7). Therefore, yoga can be said to be the path for the removal of all disturbances of the mind that cause suffering.

The path of yoga as per the *Yogasutra*, called *Ashtānga Yoga*, and also referred to as *Rāja Yoga*, involves eight limbs or stages: *yama* (ethical observances), *niyama* (self-disciplines), ā*sana* (postures), *prānāyāma* (breath regulation), *pratyāhāra* (sensory withdrawal), *dhārana* (concentration), *dhyāna* (meditation), and *samādhi* (self-transcendence). All of them are to be practiced simultaneously from the beginning to make progress on the path. The practice of these steps is expected to develop, in the practitioner, attention and awareness as a tool for discriminative knowledge (*viveka-khyāti*), which bestows the ability to perceive reality as it “truly” is, simply put, the ability to perceive phenomena without biases of any sort. The ability to see reality clearly without bias is a highly revered self-regulatory tool both within the yoga and the mindfulness traditions (Vago and Silbersweig, 2012; Gard et al., 2014). The final aim of yoga is the re-establishment of the seer or the observer within (*drashtā*) in its own form (*swarupa*) (Karambelkar, 1986, p. 9) or the realization of one's “true” self. Here, “true” has the connotation of the purest and the most pristine form of oneself, which as per yoga and Hindu philosophy is believed to be that of “total bliss.”

#### Culture as a Salient Contributor

People from different cultures vary in psychological processes—for example, fundamental processes such as attention (Miyamoto et al., 2006), and temporal ones such as daily variability in affective experiences (Oishi et al., 2004), and the studies investigating the extent of such cultural variation/similarity are of critical importance to progress in the field of psychological science (Heine and Norenzayan, 2006). Interdependent self-construals have been observed in more collectivistic Eastern cultures, where attending to others, fitting in, and harmonious relatedness with them are prioritized. On the other hand, independent self-construals have been observed in more individualistic Western cultures, wherein the priority shifts to maintaining independence from others by distinguishing oneself from others and by discovering and expressing one's unique inner attributes. The underlying cultural mechanisms of psychological processes such as interdependent vs. independent self-construals can exert a systematic influence on various fundamental aspects of cognition, emotion, and motivation (Markus and Kitayama, 1991). Culturally divergent self-construals can differentially affect other psychological processes such as inherent motivations for self-enhancement (Heine et al., 1999) and cognitive tendencies such as analytic vs. holistic thinking (Heine and Norenzayan, 2006). Furthermore, people from collectivistic cultures may be relatively more likely to attend to context than people in individualistic cultures (Chentsova-Dutton and Dzokoto, 2014). Additionally, meta-analysis has revealed the cultural differences between Asian and Western study samples in self-report measures of individualism-collectivism (Cohen's *d* of 0.39 for individualism and 0.24 for collectivism) (Oishi et al., 2004), pointing to the role of underlying culture-specific processes in the self-reports of psychological phenomena.

Scientific literature analyzing the cultural differences or similarities in the action of mind-body practices of mindfulness, particularly that of yoga, and the psychological processes underlying them is very sparse. When cultural context (Taiwanese vs. European American participants) and mindfulness were examined by Kahn et al. (2017) in relation to distress disclosure, depression symptoms, and life satisfaction, mindfulness levels moderated the relationship between distress disclosure and both depression symptoms and life satisfaction only in the Taiwanese participants but not in European Americans. This study pointed out that the benefits of disclosing distress were dependent not only on cultural context but, for the Taiwanese sample, also on mindfulness. Yoga interventions conducted as a part of research studies in India may usually contain the core practices rooted in Indian culture, for example, *mantra* chanting, which are typically not found in yoga research studies conducted in Western cultures (Wang, 2009). Thus, cultural differences either in yoga practitioners or in the effects of yoga may also emerge due to culture-specific ways of administering and absorbing yoga practices.

Cultural contexts also differ in their emphasis on foundational psychological processes, such as interoceptive awareness. Interoceptive awareness, or the awareness of bodily sensations and responsiveness to them, is one of the key abilities' yoga practices have shown to enhance (Rani and Rao, 1994; Daubenmier, 2005; Impett et al., 2006). A few studies demonstrated that people from non-Western cultures were more likely to exhibit higher levels of bodily awareness than their Western counterparts (Chentsova-Dutton and Dzokoto, 2014), with East-Asians more likely to emphasize their bodily states when describing themselves and their emotional experiences (Ma-Kellams, 2014).

Emotion regulation, in particular, and self-regulation in general, is yet another basic psychological process that is affected by culture, as also a key mechanism of action of yoga practice (Gard et al., 2014). When looking at the impact of culture on emotion regulation, Ford and Mauss (2015), in their review, suggested that culture shapes the extent to which individuals are motivated to regulate emotions, further determining the likelihood of whether emotion regulation actually takes place. They also inferred that individuals oriented toward interdependent cultural values may be more motivated to regulate their emotions by using the emotion regulation strategy of “expression suppression” as they may perceive it as adaptive based on their cultural beliefs, whereas those oriented toward independent cultural values may be less likely to do so as they may perceive this strategy as maladpative for their well-being.

Divergent self-construals in culturally diverse contexts, as mentioned earlier, are also likely to affect one's relationship with the self as well as that with others, an aspect that yoga also affects. When the relationships between self-compassion and relationally interdependent self-construal were examined, the interdependent self-construal was found to be positively related to self-kindness, common humanity, and mindfulness factors of self-compassion, and negatively correlated to self-judgment, isolation, and over-identification factors of self-compassion (Akin and Eroglu, 2013). In terms of social relatedness, a review of the evidence suggested that compared to European Americans, Asians and Asian Americans tend to shy away from explicit social support because of the concerns with potential negative relational consequences of such behavior (Kim et al., 2008).

Although the above studies make a strong case for culture as an influential variable in psychological studies, some studies have identified mindfulness as a cross-culturally similar psychological process. Ghorbani et al. (2009) found similar patterns of positive correlation of mindfulness with the self-reported adjustment comparing the participants from Iran (Middle Eastern culture) and the USA (Western culture). In yet another study, Ivtzan et al. (2018) found that after an 8-week online Mindfulness-Based Flourishing Program (MBFP), both British and Chinese (Hong Kong based) experimental groups showed significant improvements, with large effect sizes, in mindfulness, gratitude, self-compassion, meaning, and negative affect compared to a control group, providing preliminary evidence for the MBFP's cross-cultural validity. The authors concluded that the structured mindfulness-based programs lead to a similar cross-cultural pattern of benefits even when the initial understanding of mindfulness might have been different.

Despite the conflicting evidence related to culture-specific patterns in the field of mindfulness and yoga, cultural psychological research has “demonstrated that culture is implicated at a much more fundamental level of psychological processing than what was previously considered” (Heine and Norenzayan, 2006, p. 253). In fact, the inconsistent results in the above studies drive home the point that culture is an important factor to consider in psychological research, especially when culturally rooted practices, such as yoga, form a part of the research. Including culture as a factor in such psychological research will help accumulate a robust evidence-base, in turn determining the extent to which psychological processes related to mindfulness and yoga may be uniform across cultures or have culture-specific qualifiers, and thereby variations.

### The Self-Awareness, Self-Regulation, and Self-Transcendence Model

A comprehensive s*elf-awareness, self-regulation*, and *self-transcendence* (S-ART) model was developed by synthesizing the Eastern (Buddhist) traditional approaches and the Western mindfulness models with the objective of explaining the self-processing and neurological mechanisms underlying mindfulness. The S-ART model has provided an empirical framework to clarify how mindfulness reduces suffering, regulates emotions, stabilizes the mind, and develops a state of health and well-being (Vago and Silbersweig, 2012). The S-ART model has focused on two specific mindfulness meditation practices: focused-attention concentration practice and open-monitoring receptive practice (Lutz et al., 2007, 2008), and some practices to enhance ethics and develop qualities such as loving-kindness, compassion, and forgiveness (Ricard, 2003).

The S-ART model assumes that our perceptions, cognitions, and emotions are filtered by self-processing biases such as previous experiences, dispositional factors, psychological and cognitive habits, etc. These biases modify attention, perception, and cognition, “coloring” the representation of the world and of one's self, and transforming the “neutral” events into positive or negative experiences. Furthermore, affect-biased attention has been associated with affective states and symptomatology such as anxiety and depression (Yiend, 2010). In this context, mindfulness practices could generate the ability to be aware of the experiences as they genuinely are: with objectivity, and without biases and personal interpretations. According to the S-ART model, mindfulness training could reduce these biases through the development of three abilities: “*self-awareness*,” or meta-awareness of the self; “*self-regulation*,” the ability to manage responses and impulses; and “*self-transcendence*,” the expansion of the boundary of the self to include others, the development of positive relationships between self and other, and a prosocial attitude. The evolution of these three abilities modulates neurobiological changes and cognitive improvements through the integrative frontoparietal control network (Vago and Silbersweig, 2012).

As per the S-ART model, there are six neurocognitive mechanisms that underlie the abovementioned mindfulness process, which contribute to enhancing the three S-ART abilities, reducing self-biases, and maintaining a healthy mind. They are as follows:

1. *Motivation and Intention:* They are essential pillars to cultivate mindfulness. In the beginning, the motivation to do a certain practice is oriented to get some experiences and results, but with the advancement of the practice, the motivation becomes internally focused. Intention helps to follow a specific plan in the development of practice.
2. *Attention Regulation:* It is improved with the help of mindfulness practice. Advances in neuroscience research suggest a change in attention-related networks after meditative and contemplative training. Attentional effort decreases, and the efficiency of attentional networks improves over time with practice.
3. *Emotion Regulation:* Mindfulness practices also “strengthen neural systems important for emotion regulation (Vago and Silbersweig, 2012, p. 18).” Self-awareness through mindfulness practices supports the development of abilities of non-attachment and decentering. These abilities help to neutralize the negative burden of the stressor, manifesting more flexibility and openness rather than rigidity and reactivity in emotional responses. This ultimately leads to an equanimous attitude toward life situations.
4. *Extinction and Reconsolidation:* Mindfulness reduces and removes maladaptive habits and conditioning, transforming them into more positive and adaptive behaviors. The S-ART model proposes the hypothesis that this form of transformation uses the neural circuitry associated with extinction and reconsolidation. The biases of attention and memory related to habitual distortions would be extinguished and reconsolidated.
5. *Prosociality:* Mindfulness practices improve empathy. Self-awareness promotes a vision of interdependence with others, removing distinctions and boundaries between self and others. As a result, the attitudes of loving-kindness and compassion emerge in a natural way.
6. *Non-attachment and Decentering:* Awareness of the subjectivity and temporary nature of feelings, emotions, and thoughts lead to non-attachment, promoting openness and flexibility in life, and a perception of higher well-being and satisfaction with oneself and with interpersonal relationships. Decentering is defined as “the ability to observe one's thoughts and feelings as temporary, objective events in the mind, as opposed to reflections of the self that are necessarily true (Fresco et al., 2007).” Through mindfulness practice, an inner space is created in which thoughts are seen with more objectivity, and emotions, after being recognized, can be reappraised taking a wider and more expansive perspective, possibly having them lose their intensity.

### The S-ART Model in Yoga

As mentioned previously and similar to the Buddhist approach, which states, in the Four Noble Truths, that there is a way leading to the cessation of human suffering (Thera, 1993), yoga represents the pathway to the liberation of this suffering (Karambelkar, 1986). This should not be surprising as the ancient Buddhist texts and the traditional yoga texts have clear connections and mutual historical influences over time (Basavaraddi, 2015). The practice of yoga leads to an internal transformation that is reflected both in well-being in the physical, functional, emotional, cognitive, and behavioral levels as well as in interpersonal relationships and social health. Yoga builds and strengthens the internal body-mind resources with the final result of a better quality of life, which is internally based rather than externally based (Satish, 2010).

Gard et al. (2014) have proposed a theoretical framework based on an integrative systems network model to explain how yoga can optimize self-regulation through bottom-up and top-down self-regulatory processes across cognitive, emotional, behavioral, and physiological domains in the context of stress. Taking the remarkable theoretical contributions of Gard et al. (2014) and Vago and Silbersweig (2012) a step further, the current researchers propose that the common aim, connections, and mutual influences between Buddhist and yoga practices, make the S-ART model a suitable and valid theoretical framework to explain the self-regulatory mechanisms of action in yoga practice too. The following paragraphs explain the development of the three abilities of S-ART from both the theoretical and practical perspectives of yoga.

1. *Self-Awareness: Self-awareness* can be considered as the core feature of yoga practice too. Practices from various traditions of yoga including *Ashtānga Yoga* and *Hatha Yoga*, which comprise the practices of ā*sana, prānāyāma*, relaxation, meditation (*dhārana and dhyāna*), and other yoga practices, are aimed at developing an awareness of one's body, emotions, and thoughts. Through this form of body-mind training in developing awareness consciously during specific yoga practice as well as during several activities of daily life, there are greater probabilities of establishing a “witnessing self.” This “witnessing self,” referred to as the seer or the observer-within (*drashtā*) previously, confers the ability to perceive reality without biases and preconceived notions in the long run. Through the conscious cultivation of *self-awareness*, and thereby self-witnessing, yoga can lead to the integration and attunement of the organism's systemic unity encompassing the body, mind, and environment. Mindful awareness is a form of intrapersonal attunement and self-regulation. Furthermore, attunement to oneself has been proposed as the promoter of physical and psychological well-being (Siegel, 2007).
2. *Self-Regulation:* The regulation of attention, emotion, thought, action, relationships, and morality all have integration at its core (Siegel, 2019). As noted previously, yoga leads to the integration of all levels of experience (physical, emotional, and cognitive) and all aspects of human life (diet, habits, physical activity, breathing, thoughts, emotions, relationships, and ethical behavior) through practicing the path of the eight limbs of yoga as well as cultivating a healthy yogic lifestyle. This integration through yoga brings about *self-regulation*. Moreover, the current western theories of interpersonal neurobiology (Ogden et al., 2009; Siegel, 2010) have emphasized integration as a basis of well-being too.
  *Self-awareness:* is the central axis from which it is possible to integrate different levels of the human experience. This capacity of *self-awareness* to integrate aspects of the body, mind, and the environment has been proposed as the central mechanism underlying *self-regulation* and health (Siegel, 2012). Furthermore, the Western theories of the “embodied mind” (Varela et al., 1991) and the “extended mind” (Clark and Chalmers, 1998) derived from modern research in several scientific fields of psychology, linguistics, and cognitive science locate cognition as a process resulting from the interaction between the different body systems and their relationship with the environment (Garavito, 2013). These theories have also been used to explain the concept of integration in emotional processes (Barrett et al., 2005). Thus, the integration of body, mind, and their relationship with the environment can be considered as the main process underlying well-being.
  The above theories support the notion that the mind and the body are intimately related and they continuously influence each other in a circular feedback. As a result of this interaction, and thereby integration, the basic sense of being alive, wherein the experience of the body plays a fundamental role, ensues and “the physical body becomes a lived and experienced body or the basis of self-awareness” (Fuchs, 2012). Deriving from this perspective, body-awareness can be considered as the foundation of the sense of identity because it is the consciousness of the body that would generate the awareness of itself. In other words, the consciousness of the body (body-awareness) itself is the foundation of *self-awareness*.
  An example of how therapy can be based on body-awareness is provided by the “model of sensorimotor therapy” (Ogden et al., 2009), wherein the integration of all levels of experience—sensorimotor, emotional, and cognitive—starts with an awareness of body sensations. In the same way, starting from a visceral, sensorial and motor awareness, and through the development of “bottom-up” as well as “top-down” awareness of emotional and cognitive processes, yoga practices act on all levels of experience-organization (physical, emotional and cognitive), facilitate their integration, and thereby bring about self-regulation and well-being. Supporting this idea is the previously cited study of Gard et al. (2014) regarding the potential self-regulatory mechanisms of yoga for psychological health. The authors posit that yoga practices would lead to self-regulation through both the promotion of behavioral changes as well as the inhibition of a non-desirable output by both higher-level and lower-level brain networks when facing stress-related challenges, with a consequent positive impact on health and well-being. In their review, the authors also provided sufficient empirical evidence for the association between yoga and self-regulation across cognitive, behavioral, autonomic, and emotion domains.
3. *Self-Transcendence:* The final step (i.e., aim) of *Asthānga Yoga* is a state of *self-transcendence*, and all its preceding steps facilitate the accomplishment of this final aim. The first two limbs in *Asthānga Yoga* of *yama and niyama*, the observances related to social and personal conduct, respectively, lead to the regulation of aspects related to the self and others. The five *yama*, as per the *Yogasutra*, of *ahimsa* (nonviolence), *satya* (truthfulness), *asteya* (non-stealing), *brahmacharya* (right use of energy), and *aparigraha* (non-hoarding) help to regulate one's behavior and promote adherence to ethical values in the society at large. The five *niyama* as per the *Yogasutra* of *shaucha* (cleanliness), *santosha* (contentment), *tapas* (discipline and austerity), *swādhyāya* (study of the self and of the sacred texts), and *ishvara-pranidhāna* (surrender to or contemplation of a higher power) all help to discipline, calm down, and pacify the mind.
  The next three limbs, after *yama and niyama*, of ā*sana* (steady and comfortable bodily postures), *prānāyāma* (voluntary regulation of breath), and *pratyāhāra* (inward withdrawal of the senses) relate to working with one's physical, physiological, and psychological aspects with conscious awareness and regulating those levels of experience in a stepwise manner. The last three limbs of *dhārana, dhyāna*, and *samādhi* involve working with the subtler and more spiritual aspects of the mind, by taking the inward withdrawal in *pratyāhāra* to higher and more refined levels, and honing one's degree of attention step-by-step through building concentration (*dhārana)*, attaining deeper meditative states (*dhyāna*), and, finally, transcending the self (*samādhi*).
  The *Yogasutra* also provides a practical and powerful technique to discipline oneself to have a calm mind during social behavior. This is the technique of “*citta-prasādana,”* which is mentioned in the 33rd verse of its first chapter. From the assumption that the mind gets disturbed by the existence of negative attitudes, feelings, and thoughts in relation to others, the cultivation of specific attitudes—of friendship toward happy people, of compassion toward people who undergo suffering, of joy toward others virtues, and of emotional distance toward the harmful attitudes of others, as well as, the repeated application of these attitudes—are prescribed as one of the most powerful ways to achieve “*citta-prasādana*,” or a peaceful state of mind (Karambelkar, 1986).
  As explained previously, integration is not only the core of *self-regulation*, but also the main process underlying well-being too. *Self-awareness* fosters the integration of an individual's mind, brain, and body, and compassion promotes the integration of individuals in their relationships, in the family and the society. According to Siegel (2017), compassion (the feeling to want to help others), connection (the awareness of being linked with others), equanimity (the regulation of one's own internal state), which in turn allow to maintain compassion and the sense of connection, and a sense of higher meaning or purpose (the intention of contributing with others, the society, and the world in large), are all positive and healing capacities of the mind related to integration.
  The systematic observance of the eight limbs of *Asthānga Yoga* as well as the method of “*citta-prasādana*,” leads to a state of equilibrium in the body-mind complex, peace, and clarity of mind, and ultimately, to illumination or real knowledge of the unity of existence both within and outside. When the practitioner achieves this enlightenment or “self-realization,” as it is called in yoga, it leads to the realization that there is no difference between the self and the other, thereby generating a natural feeling of love toward every living creature. This is the accomplishment of *self-transcendence*.

### Perseverance in Yoga Practice

The significance of the maintenance of yoga practice for accomplishing complete tranquility of mind can be observed in the *Yogasutra*. For example, the 12th and the 13th verse from the first chapter of the *Yogasutra* emphasize the significance of repeated yoga practice in order to achieve steadiness in a state of freedom from those *vrtti* (functional modifications of the mind; Karambelkar, 1986), which apparently prevent one from experiencing the moment as it is. The next verse (14th) from the same chapter clearly states that practice gets firmly grounded when it has been adopted for a long duration of time, in an uninterrupted manner, and with a receptive attitude (Karambelkar, 1986). Furthermore, as per the *Yogasutra*, in order to achieve a peaceful mind through the cultivation of the suggested attitudes of “*citta-prasādana*,” mentioned earlier, they need to be repeated and sustained over time for reversing the negative tendencies of the mind.

The degree of accomplishment in yoga also depends on the intensity of the yoga practitioner's urge (*Yogasutra*-−1st chapter, 21st *sutra*). Furthermore, even in those with intense urge, the *Yogasutra* has mentioned three categories of yoga practitioners (*mrudu*—mild, *madhya*—medium, and *adhimātra*—extraordinary) depending on the efforts taken and the commitment toward yoga practice (*Yogasutra*-−1st chapter, 22nd *sutra*). Thus, it is apt to conclude that the *Yogasutra* has laid a special emphasis on the significance of sustained and committed practice of the eight limbs of *Ashtānga Yoga* for achieving discriminative knowledge (*viveka-khyāti*) (*Yogasutra*-−2nd chapter, 28th *sutra*; Karambelkar, 1986), the meta-ability to have an overall unbiased perception of phenomena.

Research studying the direct contribution or influence of perseverance in yoga practice on various aspects of well-being is, as yet, few and far between. One of the few studies directly measuring this to some extent is a single-arm study of a 2-month yoga immersion program with 19 participants, which demonstrated that higher amounts of yoga practice were associated with increased body awareness, positive affect, and satisfaction with life, as well as decreased negative affect for both women and men (Impett et al., 2006).

### Overview of the Present Study

Our study had two main objectives and one exploratory objective. The first main objective of the current study, in line with the arguments made in the section Introduction, was to build on the S-ART model, and propose it as a theoretical framework to explain the self-regulatory mechanisms of action in yoga practice.

As its second main objective, the current study aimed at producing preliminary empirical evidence for the S-ART model in yoga by investigating the action of yoga practice on the psychological variables mapped to the S-ART foundational abilities of *self-awareness, self-regulation*, and *self-transcendence* in a sample of yoga practitioners and non-practitioners (NP). Although some evidence exists on the role of yoga in relation to each of these S-ART meta-abilities, our study will be the first to study all of them comprehensively at one time within a sound theoretical framework, which is expected to produce some novel insights.

Evidence related to the action of yoga in interoceptive awareness has already been cited earlier. Interoceptive awareness can be considered as a key starting point to activate *self-awareness* mechanisms. Also, an awareness of interoceptive experience facilitates the integration across various layers of human functioning, and yoga offers a way to practice this integration, which can also be considered as a form of self-regulation (Gard et al., 2014).

Regarding evidence related to the second S-ART meta-ability of self-regulation, in a review of 24 articles on the emotion regulation potential of yoga practice, Menezes et al. (2015) found evidence of the effect of yoga in the improvement of emotional functioning in both healthy subjects and people who suffer from different health conditions. This evidence suggests that yoga can help to promote healthier psychological responses and it is a potential emotion regulation strategy working through mechanisms such as reappraisal, attention regulation, self-monitoring, self-awareness, and autonomic regulation. Gard et al. (2014), based on evidence reviewed from various studies, in which yoga aided and promoted self-regulation across cognitive, emotional, behavioral, and autonomic domains, proposed a model for the self-regulatory mechanisms of yoga in well-being. Previous research by the first author of the current study also explored the role of yoga on self-regulation, showing that practicing yoga had a beneficial effect on physiological, emotional, and cognitive self-regulation in patients who suffered from essential arterial hypertension (Tolbaños Roche et al., 2017).

Although there is little evidence addressing the role of yoga in *self-transcendence*, some studies have demonstrated preliminary results for a higher level of this third S-ART meta-ability in regular yoga practitioners, compared to NP (Fiori et al., 2014) and also after a single session of yoga (Park et al., 2020), as well as in prison inmates (Griera, 2017). A few studies have also demonstrated preliminary positive results for the social connectedness aspect of yoga (see Park et al., 2020). As *self-transcendence* is also considered as the social component related to yoga practice, which helps to promote interdependence (Gard et al., 2014), these studies on social connectedness tend to support the case for psychological mechanisms of *self-transcendence* in yoga.

Previous evidence has shown positive results with yoga practice in shorter interventions. However, some of the studies have remarked the importance of regularity of practice, demonstrating that more practice was associated with lower mood disturbances (Khalsa et al., 2012), cognitive emotion regulation (Gootjes et al., 2011), and greater well-being and quality of sleep (Danhauer et al., 2009). Therefore, as a part of its second objective, our study aimed at studying how perseverance in yoga practice (long-term yoga practice) further contributed to the self-regulatory mechanisms of action in yoga.

There remains a substantive gap in our knowledge about how Western models of psychological processes, such as body consciousness, emerge—or fail to emerge—in other cultural contexts (Ma-Kellams, 2014). Moreover, we also found a lack of adequate studies looking at the role of different cultural contexts in yoga, particularly related to that of the self-regulatory action in yoga practice. Therefore, we thought that it is important to explore the role of yoga, within the S-ART model, in two culturally diverse contexts—that of the collectivistic Eastern culture of India and that of the individualistic Western culture of Spain. This formed part of our third exploratory objective, which could possibly help to generate worthwhile information for the formulation of hypotheses in future research.

Based on the evidence cited above, we formulated two hypotheses in relation to the second objective of the study. We expected higher levels of S-ART in yoga practitioners compared to NP, as well as in longer-term practitioners. We did not formulate any hypothesis related to the third objective regarding the role of yoga in two culturally diverse samples as our study was an exploratory study in this regard.

Each of the three abilities of the S-ART model was appropriately mapped to four psychological variables of interoceptive awareness (multidimensional), decentering, emotion regulation, and relational compassion, with the middle two variables used as indicators of self-regulation. These model-mapped variables were treated as our outcome psychological variables, and were measured by using the standard psychological instruments elaborated in the next section. Very few studies have attempted to take a multidimensional view of variables such as somatic awareness (one of the key skills implicated in interoceptive awareness) when dealing with different cultural contexts. Given the need for a greater precision in defining the nature of observed cultural variations, research would benefit with a more systematic use of multidimensional measures, for example, the Multidimensional Assessment of Interoceptive Awareness (MAIA; Mehling et al., 2012), in culturally diverse contexts (Ma-Kellams, 2014). In line with this suggestion, our study attempted to take a multidimensional view of the S-ART meta-abilities when selecting the psychological instruments to measure them.

## Methods

### Participants

The total sample in this cross-sectional study consisted of 362 participants (197 Indian and 165 Spanish) of both genders (98 males and 264 females), between 17 and 77 years old. The mean age was 40.27 (SD 14.06). Of the total sample, 232 participants were yoga practitioners and 125 participants were NP, with a larger proportion of the Indian sample being practitioners (147, 76.56%) compared to NP (45, 23.4%) whereas the Spanish sample being more equally divided between practitioners (85, 51.52%) and NP (80, 48.48%). The Indian sample of yoga practitioners as well as NP (never or <1 month of yoga practice) was constituted by the students enrolled in the short-term certificate yoga courses (1–3 months) conducted through the Mumbai, Pune, and Lonavala branches/collaborative centers of a traditional yoga institute in Western India, life members and regular participants taking yoga classes at the Mumbai health center of the said yoga institute, participants visiting the Lonavala health center of the said yoga institute for 1–3 weeks of health and rejuvenation programs, as well as employees of the said yoga institute from their Mumbai and Lonavala locations. The Spanish sample of yoga practitioners was composed mainly of the participants from the regular yoga classes at a yoga studio located at Las Palmas, Canary Islands, Spain, whereas the NP were mainly the students of Medicine and Psychology coming from the three different national universities located in the mainland of Spain and one national university located in Tenerife, Spain.

The aforementioned yoga institute in India and the yoga studio in Spain, from where mainly the yoga practitioner data were collected, both taught traditional *Ashtānga Yoga*, and encouraged its participants and students to practice all the yoga practices of ā*sana, prānāyāma*, relaxation, and meditation every day. Many participants of the study combined the studies with their professional careers or other occupations. Apart from collecting data about the study variables, we also gathered sociodemographic data and information related to the participant's yoga practice.

### Instruments

The standard psychological instruments used in the current study were used to measure the dependent psychological variables mapped to the aforementioned three S-ART abilities. The six neurocognitive mechanisms underlying the mindfulness process in the framework of the S-ART model formed a basis for the selection of these psychological instruments.

The original English and the validated Spanish version of the MAIA (Mehling et al., 2012; Valenzuela Moguillansky and Reyes-Reyes, 2015) were used to measure *self-awareness* in terms of interoceptive awareness, attention, emotional awareness, and to measure *self-regulation*. This is a 32-item multidimensional instrument with eight subscales of three to seven items each. The subscales are: Noticing, Not-distracting, Not-Worrying, Attention Regulation, Emotional Awareness, Self-Regulation, Body Listening, and Trusting. For this scale, Cronbach's alphas in the range between 0.66 and 0.82 have been obtained.

Although two of the MAIA subscales of attention regulation and self-regulation do assess *self-regulation*, it is assessed mainly in terms of the ability to sustain and control attention to body sensations. Moreover, the main aim of these two subscales is the assessment of body awareness. Therefore, we decided to use additional scales assessing other aspects of *self-regulation*, such as the cognitive ability of decentering, the difficulties encountered when regulating emotion, or emotion regulation deficit.

The original English and the validated Spanish version of the Decentering subscale of the Experiences Questionnaire-Decentering (Fresco et al., 2007; Soler et al., 2014; EQ-D) were used to measure *self-regulation* in terms of decentering ability. Decentering is defined as the ability to observe one's thoughts and feelings in a detached manner. The EQ-D subscale contains 11 items, with a Cronbach's alpha of 0.89.

The English Brief Version of the Difficulties in Emotion Regulation Scale (DERS; Victor and Klonsky, 2016) and the Spanish translation (acquired by the *Computer Assisted Culturally Informed and Flexible Family Based Treatment for Adolescents* study team of the University of Miami) were also used to measure *self-regulation* through the evaluation of difficulties in emotion regulation. This 18-item scale is composed of the strongest items from each of the six subscales from the original DERS publication (Gratz and Roemer, 2004), with subscale alphas ranging from 0.77 to 0.90 and an alpha of 0.91 for the entire scale. The test subscales are: Awareness, Clarity, Goals, Impulse, Non-acceptance, and Strategies.

The original English and the validated Spanish version of the Relational Compassion Scale (RCS; Hacker, 2008; Garcia-Campayo et al., 2014) were used to measure *self-transcendence* in terms of the development of positive relationships between self and other, and prosocial abilities. This 16-item scale comprises four subscales: self-self, others-self, self-others, others-others with the Cronbach's alpha for the subscales ranging from acceptable to good: 0.74–0.84.

### Procedure

The data in the current study were obtained through convenient purposive sampling; although we targeted yoga institutes and educational set-ups, it was more of a convenience sample. For the Indian sample, the test administration was done in person with paper–pencil tests, mostly in groups at predecided times after explaining the purpose of the study and obtaining a verbal and written consent of participation. The Spanish participants were asked to respond to the study questionnaires in an electronic format through Google forms, which included detailed information about the study and informed consent.

Although no formal record of response rate was maintained, on an average, we can say that for both the Indian and Spanish samples, it was above 90% as tests were provided to the participants only after obtaining the verbal consent for participation in the study and adequate reminders were given until form completion. There were missing data from five participants in the total sample.

### Data Analyses

The statistical package SPSS, version 15.0, was used for conducting the data analysis. A one-way ANOVA for the numerical variables and the chi-squared tests for the categorical variables were used for analyzing the homogeneity of the two groups of yoga practitioners and NP in sociodemographic characteristics of the total sample, life habits, and different aspects related to the practice of yoga. After this preliminary analysis, first, the Pearson correlation analysis was conducted to examine which of the measured sociodemographic characteristics, life habits, and aspects of yoga practice were significantly correlated with the four psychological outcome variables of Interoceptive Awareness, Decentering, Difficulties in Emotional Regulation (DERS), and Relational Compassion, as measured by MAIA, EQ-D, DERS, and RCS, respectively. Second, a one-way Multivariate ANOVA (MANOVA) was employed to test our first hypothesis and to determine whether there were differences between yoga practitioners and NP on the total scores of the outcome variables.

Next, we tested our second hypothesis related to the contribution of perseverance in yoga practice, measured by the length of yoga practice in months/years, on the psychological outcome variables, which showed significantly higher levels in yoga practitioners in the first hypothesis testing. The hierarchical regression analysis was conducted to explore the independent and additive power of perseverance in yoga practice when sociodemographic characteristics, life habits, and different aspects of yoga practice, which showed a significant correlation with these selected outcome variables, were controlled. In this regression analysis, the yoga practitioners were categorized into three groups: beginners (BG; between 1 month and <1 year of practice), medium-term practitioners (MP; between 1 and 5 years of practice), and long-term practitioners (LP; >5 years of practice).

Finally, we addressed our third objective of exploring the specific self-regulatory contribution of yoga in each cultural sample of Indian and Spanish participants. In order to fulfill this objective, first, the Pearson correlation analysis was conducted to examine which of the measured sociodemographic characteristics, life habits, and aspects of yoga practice in each of the Indian and Spanish samples were significantly associated with the four psychological outcome variables. Next, a one-way MANOVA was performed to determine whether there were differences between yoga practitioners and NP, on the total scores of the four outcome variables. In this MANOVA, the participants of each cultural sample were divided into three groups of NP (never or <1 month of yoga practice), BG (1 month to 1 year of yoga practice), and above 1- year practitioners (AYP; >1 year of yoga practice), so as to get further exploratory information on how yoga practice as well as perseverance in it would act on the outcome variables. As per the results obtained from this one-way MANOVA analysis on the total scores of the outcome variables, we conducted an exploratory principal components analysis (PCA) with Varimax rotation (extraction criterion: eigenvalue>1) only on the MAIA scale for each cultural sample. This exploratory PCA was done in order to examine whether the component/factor structure of the original MAIA version, represented by its subscales, would reproduce in both the Indian and Spanish samples, and in the event of divergence, to determine the MAIA component/factor structure of each cultural sample, enabling a more detailed examination of the culture-specific grouping of skills represented by its component subscales. Cronbach's alpha coefficient was used to assess the internal consistency reliability of the full MAIA scale in the total sample, as well as in the full MAIA scale and in its components (obtained from PCA) at each cultural sample. We also performed a hierarchical regression analysis in order to explore the independent and additive power of the practice of yoga and perseverance in it on the relevant outcome variables when sociodemographic characteristics, life habits, and different aspects of yoga practice, significantly correlated with these outcome variables, were controlled.

## Results

### Preliminary Analyses

The descriptive statistics and the statistical significance of the differences between yoga practitioners and NP in sociodemographic variables (age, gender, marital status, education, and occupation), health and life habits (weight, height, smoking, drinking, and the frequency of physical exercise), and the differences in percentages among yoga practitioners on aspects of yoga practice [the type/style of yoga, frequency of yoga practice, type of (specific) yoga practices, and length of practice] are shown in Tables 1A–C, respectively.

**Table 1A:** Descriptive statistics and differences between yoga practitioners and non-practitioners (NP) in sociodemographic variables.

		**Practitioners**	**Non-practitioners**	**χ^2^/F**	***p***
		**(*N* = 232)**	**(*N* = 125)**		
Age(M ± SD)	Total	41.62 ± 13.18	37.82 ± 14.51	5.813	0.016
	Females	41.27 ± 13.12	37.12 ± 14.75		
	Males	42.60 ± 15.58	39.60 ± 13.90		
Gender (%)				0.151	0.697
	Females	73.9%	72.0%		
	Males	26.1%	28.0%		
Nationality (%)				24.467	<0.001
	Indian	76.6% (N 147)	23.4% (N 45)		
	Spanish	51.5% (N 85)	48.5% (N 80)		
Marital status (%)				7.775	0.169
	Single	40.9%	52.4%		
	Married	49.8%	40.3%		
	Separed	1.3%	3.2%		
	Divorced	4.9%	3.2%		
	Widow/er	2.7%	0.8%		
	Others	0.3%	0.0%		
Education (%)				25.103	<0.001
	Primary	0.0%	1.8%		
	Middle	6.1%	17.3%		
	Diploma	19.2%	25.5%		
	Graduate	33.8%	37.3%		
	Postgraduate	40.9%	6.5%		
Occupation (%)				13.093	0.042
	Employed	62.7%	37.3%		
	Business	9.8%	5.7%		
	Freelancer	11.6%	5.7%		
	Student	18.2%	30.1%		
	Homemaker	15.1%	8.9%		
	Retired	5.8%	4.9%		
	Unemployed	2.2%	4.1%		

**Table 1B:** Descriptive statistics and differences between yoga practitioners and non-practitioners in weight, height, and life habits.

		**Practitioners**	**Non-practitioners**	**χ^2^/F**	***p***
		**(*N* = 232)**	**(*N* = 125)**		
Weight(M ± SD)	Total	65.01 ± 12.19	66.73 ± 13.17	1.479	0.225
	Females	61.82 ± 10.88	62.38 ± 11.37		
	Males	73.93 ± 11.26	78.81 ± 9.94		
Height(M ± SD)	Total	163.39 ± 11.30	164.68 ± 16.04	0.773	0.380
	Females	160.04 ± 10.53	161.47 ± 17.23		
	Males	172.46 ± 7.80	173.37 ± 6.78		
Smoking (%)				5.123	0.024
	No	95.7%	88.2%		
	Yes	4.26%	11.8%		
Alcohol (%)				10.021	0.018
	Never	53.0%	36.0%		
	Occasionally	42.2%	57.6%		
	Frequently	2.2%	4.00%		
	Regularly	2.6%	2.4%		
Physical exercise (%)				18.103	<0.001
	None	5.8%	15.0%		
	Low	26.3%	38.3%		
	Moderate	62.1%	40.0%		
	High	5.8%	6.7%		

**Table 1C:** Differences in percentages among yoga practitioners on aspects of yoga practice.

		**Practitioners**
		**(*N* = 232)**
Type of yoga (%)	Traditional	84.4%
	Modern	11.8%
	Both	3.8%
Frequency of practice (%)	Once	12.6%
	Twice	21.7%
	Thrice	15.2%
	Four times	9.7%
	Five times	16.1%
	Six times	10.4%
	Everyday	14.3%
Type of practices (%)	Asana	8.0%
	Pranayama	1.8%
	Yogic relaxation	1.3%
	Meditation	3.1%
	More than one	40.0%
	All	45.8%
Lenght of practice (%)	Beginners	31.4%
	Medium-term	45.8%
	Long-term	23.8%

*Length of practice—beginners (BG; 1 month to <1 year of practice), medium-term practitioners (MP; 1–5 years of practice), and long-term practitioners (LP; >5 years of practice)*.

### Main Analyses

#### Correlations of the Outcome Variables With Sociodemographic Characteristics, Life Habits, and Aspects of Yoga Practice

When the association of the four outcome variables with the sociodemographic characteristics, life habits, and aspects of yoga practice was analyzed, the results, shown in Table 2, indicated significant correlations of Interoceptive Awareness (MAIA) with marital status, physical exercise, frequency of practice, and type of (yoga) practices, that of decentering (EQ-D) with gender, education, weight, physical exercise, and frequency of practice, that of DERS with age, and physical exercise, and that of RCS with physical exercise.

#### Differences Between Yoga Practitioners and NP in the Outcome Variables Scores

When testing our first hypothesis related to the differences between yoga practitioners and NP in the outcome variables, the results of MANOVA showed a significant multivariate effect on the total scores of the outcome variables of MAIA, EQ-D, DERS, and RCS (*F* = 6.305; *p* < 0.001; *df* = 4; power = 0.989). The tests of between-subject effects reported statistically significant differences between yoga practitioners and NP in the MAIA (*F* = 24.457; *p* < 0.001; *df* = 1; power = 0.999) and the EQ-D scores (*F* = 12.143; *p* = 0.001; *df* = 1; power = 0.935).

According to the findings mentioned above, yoga practitioners showed significantly higher interoceptive awareness and significantly higher decentering ability than NP. Thus, in support of the first hypothesis, the yoga practitioners in the current study demonstrated better self-awareness and self-regulatory abilities (as indicated by the total scores on MAIA and EQ-D, respectively).

#### Contribution of Perseverance in Practice of Yoga Practitioners on the Outcome Variables

The results of the hierarchical regression analysis, shown in Table 3, indicated that perseverance in yoga practice (as measured by the length of yoga practice) acted as a significant contributor of the total MAIA score after controlling those sociodemographic characteristics (marital status), life habits (physical exercise), and aspects of yoga practice (frequency of yoga practice, and type of practices) that showed a significant correlation with MAIA. Likewise, perseverance in yoga practice significantly contributed to the total EQ-D score after controlling those sociodemographic characteristics (gender, education, and weight), life habits (physical exercise), and aspects of yoga practice (frequency of yoga practice) that were significantly associated with EQ-D.

The abovementioned results from the regression supported the second hypothesis to a reasonable extent, indicating that perseverance in yoga practice acted as a significant predictor of interoceptive awareness, a vital aspect of *self-awareness*, and self-regulatory abilities, in the yoga practitioners of the current study.

#### Additional Analyses: Exploration of the Contribution of Yoga Practice in Each Cultural Sample of Indian and Spanish Participants

##### Correlations of the Outcome Variables in Each Cultural Sample With Sociodemographic Characteristics, Life Habits, and Aspects of Yoga Practice

When the association of the four outcome variables with sociodemographic characteristics, life habits, and aspects of yoga practice were examined for each cultural sample (indicated by nationality), the results related to the Indian participants, shown in Table 4, indicated that Interoceptive Awareness (MAIA) was significantly correlated with smoking and physical exercise, and decentering (EQ-D) with height and physical exercise.

**Table 2:** Bivariate correlations of the outcome variables with socio-demographic characteristics, life habits and aspects of yoga practice.

	**Gender**	**Age**	**Nationality**	**Marital**	**Educ**.	**Occup**.	**Weight**	**Height**	**Smoking**	**Alcohol**	**Physical**	**Yoga**	**Practice**	**Type of**
				**status**							**exercise**	**type**	**freq**.	**practices**
MAIA	−0.05	0.04	−0.07	0.11^*^	0.09	0.04	0.01	−0.01	−0.09	−0.09	0.20^**^	0.11	0.14^*^	0.14^*^
EQ-D	−0.18^**^	0.07	−0.08	0.09	0.14^*^	−0.00	0.11^*^	0.10	0.02	−0.07	0.18^**^	0.12	0.18^**^	0.11
DERS	0.05	−0.12^*^	−0.06	−0.09	−0.00	0.03	−0.08	−0.07	−0.06	0.05	−0.13^*^	−0.02	−0.02	−0.08
RCS	−0.04	0.00	0.07	0.03	0.05	0.02	0.06	0.02	−0.03	0.02	0.12^*^	0.10	0.05	0.00

** *p < 0.01;*

* *p < 0.05;*

*Educ., Education; Occup., Occupation; Practice Freq., Frequency of practice*.

**Table 3:** Hierarchical regression analysis with perseverance in yoga as the predictor variable in total Multidimensional Assessment of Interoceptive Awareness (MAIA) and Experiences Questionnaire-Decentering (EQ-D) total scores after controlling sociodemographic characteristics, life habits, and aspects of yoga practice.

	**MAIA**					**EQ-D**				
	**B**	**SE B**	**β**	**t**	***p***	**B**	**SE B**	**β**	**T**	**p**
Gender	–	–	–	–	–	−3.267	1.159	−0.233	−2.819	0.005
Education	–	–	–	–	–	−3.374	0.540	−0.055	−0.693	0.489
Marital status	2.105	1.518	0.097	1.387	0.167	–	–	–	–	–
Weight	–	–	–	–	–	0.026	0.042	0.050	0.615	0.540
Physical exercise	5.869	2.280	0.179	2.574	0.011	1.702	0.725	0.174	2.346	0.020
Frequency of practices	1.346	0.745	0.131	1.807	0.072	0.528	0.251	0.167	2.102	0.037
Type of practices	1.099	0.827	0.092	1.329	0.185	–	–	–	–	–
Length of practice	6.093	2.040	0.211	2.987	0.003	1.340	0.665	0.149	2.104	0.046

*Length of practice (perseverance in yoga) was categorized into three groups of BG (1 month to <1 year of practice), MP (1–5 years of practice), and LP (>5 years of practice)*.

**Table 4:** Bivariate correlations of the outcome variables with sociodemographic characteristics, life habits, and aspects of yoga practice in the Indian participants.

	**Gender**	**Age**	**Marital**	**Education**	**Occupation**	**Weight**	**Height**	**Smoking**	**Alcohol**	**Physical**	**Type of**	**Practice**	**Type of**
			**status**							**exercise**	**yoga**	**frequency**	**practices**
MAIA	−0.00	0.06	0.06	0.13	−0.08	0.03	0.03	−0.16^*^	−0.08	0.20^**^	0.05	−0.00	0.15
EQ-D	−0.11	0.04	0.04	0.07	−0.13	0.12	0.18^*^	0.07	−0.05	0.19^*^	0.07	0.03	0.08
DERS	0.10	−0.06	−0.06	−0.10	0.13	−0.01	0.03	−0.00	0.09	−0.14	0.02	0.012	−0.02
RCS	0.02	0.08	0.08	0.09	0.02	0.12	0.05	−0.05	0.01	0.13	0.08	−0.04	0.07

** *p < 0.01;*

* *p < 0.05*.

The results related to the Spanish participants, shown in Table 5, revealed significant correlations of Interoceptive Awareness (MAIA) with physical exercise, type of yoga, and frequency of yoga practice, that of decentering (EQ-D) with gender, smoking, physical exercise, and type of yoga, that of DERS with gender, age, weight, frequency of practice, and type of practices, and that of RCS with the frequency of practice.

**Table 5:** Bivariate correlations of the outcome variables with sociodemographic characteristics, life habits, and aspects of yoga practice in the Spanish participants.

	**Gender**	**Age**	**Marital**	**Education**	**Occupation**	**Weight**	**Height**	**Smoking**	**Alcohol**	**Physical**	**Type of**	**Practice**	**Type of**
			**status**							**exercise**	**yoga**	**frequency**	**practices**
MAIA	−0.09	0.03	0.16^*^	−0.09	0.12	−0.00	−0.02	0.08	−0.06	0.20^*^	0.23^*^	0.40^**^	0.13
EQ–D	−0.25^**^	0.06	0.14	0.12	0.09	0.10	0.07	0.23^*^	−0.03	0.17^*^	0.25^*^	0.43^**^	0.14
DERS	0.27^**^	−0.17^*^	−0.11	−0.03	−0.08	−0.18^*^	−0.14	−0.10	0.09	−0.13	−0.15	−0.21^*^	−0.22^*^
RCS	−0.12	−0.12	−0.01	0.05	0.05	−0.03	−0.00	0.03	−0.03	0.12	0.18	0.31^**^	−0.07

** *p < 0.01;*

* *p < 0.05*.

##### Differences Among NP, BG, and AYP on the Total Scores of the Outcome Variables and the Results of the Hierarchical Regression Analysis in Each Cultural Sample

The results of MANOVA showed a statistically significant multivariate effect on the total scores of MAIA, EQ-D, DERS, and RCS in the Indian participants (*F* = 2.530; *p* = 0.011; *df* = 8; power = 0.912). The tests of between-subjects effects displayed statistically significant differences among NP, BG, and AYP in the MAIA (*F* = 9.312; *p* < 0.001; *df* = 2; power = 0.976), and EQ-D scores (*F* = 6.384; *p* = 0.002; *df* = 2; power = 0.897). The results of the multiple comparisons (Table 6) showed significantly higher total scores on MAIA and EQ-D of only AYP in the Indian participants, indicating the influence of perseverance in yoga practice on the abilities of interoceptive awareness (*self-awareness*) and decentering (*self-regulation*) of Indian participants in the current study.

**Table 6:** Multiple comparisons between groups based on yoga practice in total MAIA and EQ-D scores in the Indian participants.

	**(I)**	**(J)**	**Mean difference (I – J)**
MAIATOT	NP	BG	−6.62
		AYP	−15.73^*^
	BG	NP	6.62
		AYP	−9.11^*^
	AYP	NP	15.73^*^
		BG	9.11^*^
EQTOT	NP	BG	−2.75
		AYP	−4.17^*^
	BG	NP	2.75
		AYP	−1.42
	AYP	NP	4.17^*^
		BG	1.42

* *p < 0.05,*

*NP, Non-practitioners; BG, Beginners; AYP, Above 1-year practitioners*.

The results of the hierarchical regression analysis, shown in Table 7, indicated that the practice of yoga and perseverance in it acted as a significant contributor of the total MAIA score in the Indian participants after controlling those life habits (smoking and physical exercise) that were significantly correlated with MAIA. Likewise, yoga practice and perseverance in it significantly contributed to the total EQ-D score after controlling those sociodemographic characteristics (height) and life habits (physical exercise) that were significantly associated with EQ-D. These results showed that the practice of yoga and perseverance in it acted as a significant predictor of the abilities of interoceptive awareness and decentering in the Indian participants of the current study.

A statistically significant multivariate effect of MANOVA on the total scores of MAIA, EQ-D, DERS, and RCS in the Spanish participants was found (*F* = 3.476; *p* = 0.001; *df* = 8; power = 0.980). The tests of between-subjects effects displayed statistically significant differences among NP, BG, and AYP in MAIA (*F* = 9.782; *p* < 0.001; *df* = 2; power = 0.982) and in EQ-D (*F* = 8.352; *p* = 0.002; *df* = 2; power = 0.961).

**Table 7:** Hierarchical regression analysis with practice of yoga and perseverance in it as the predictor variable in total MAIA and EQ-D scores of the Indian participants after controlling sociodemographic characteristics, life habits, and aspects of yoga practice.

	**MAIA**					**EQ-D**				
	**B**	**SE B**	**β**	**t**	***p***	**B**	**SE B**	**β**	**t**	***p***
Smoking	−15.719	6.836	−0.168	−2.300	0.023	–	–	–	–	–
Height	–	–	–	–	–	0.132	0.047	0.209	2.808	0.006
Physical exercise	3.484	1.962	0.134	1.174	0.078	1.557	0.605	0.198	2.575	0.011
Practice/length of yoga	6.883	1.889	0.275	3.644	<0.001	1.523	0.572	0.205	2.663	0.009

*Practice/length of yoga (yoga practice and perseverance in it) was categorized into three groups of NP (never or <1month of yoga practice), BG (1 month to <1 year of practice), and AYP (>1 year of practice)*.

However, the results of the hierarchical regression analysis, shown in Table 8, indicated that, after controlling those sociodemographic characteristics (marital status), life habits (physical exercise), and aspects of yoga practice (type of yoga and frequency of practice) that were significantly correlated with the total MAIA score, the practice of yoga and perseverance in it were not its (of MAIA) significant contributors in the Spanish participants. Likewise, after controlling those sociodemographic characteristics (gender), life habits (physical exercise and smoking), and aspects of yoga practice (type of yoga and frequency of practice) that were significantly correlated with the total EQ-D score, yoga practice and perseverance in it did not significantly contribute to it. A detailed look at the regression results indicated that, in the Spanish participants of the current study, physical exercise and frequency of yoga practice emerged as better predictors of the abilities of interoceptive awareness (*self-awareness*) and decentering (*self-regulation*) than yoga practice and perseverance in it.

**Table 8:** Hierarchical regression analysis with practice of yoga and perseverance in it as the predictor variable in total MAIA and EQ-D scores of the Spanish participants after controlling sociodemographic characteristics, life habits, and aspects of yoga practice.

	**MAIA**					**EQ-D**				
	**B**	**SE B**	**β**	**t**	***p***	**B**	**SE B**	**β**	**t**	***P***
Gender	–	–	–	–	–	0.990	3.317	0.049	0.298	0.767
Marital status	2.320	1.913	0.122	1.213	0.229	–	–	–	–	–
Smoking	–	–	–	–	–	−1.520	3.154	−0.085	−0.482	0.633
Physical exercise	9.376	4.319	0.226	2.171	0.033	1.215	1.820	0.113	0.668	0.509
Type of yoga	−0.922	7.039	−0.014	−0.131	0.896	0.787	3.926	0.032	0.200	0.842
Frequency of practices	4.281	1.494	0.298	2.864	0.005	1.343	1.056	0.205	1.271	0.213
Practice/length of yoga	7.591	4.425	0.175	1.716	0.090	1.951	1.495	0.243	1.306	0.201

*Practice/length of yoga (yoga practice and perseverance in it) was categorized into three groups of NP (never or <1 month of yoga practice), BG (1 month to <1 year of practice), and AYP (>1 year of practice)*.

#### Exploratory Factor Analysis of the MAIA Scale in Each Cultural Sample

It was important to understand the similarity or difference of factor structure of the MAIA scale in each cultural sample as it emerged as a significant outcome variable in the aforementioned MANOVA analysis in both the Indian and Spanish samples.

In the exploratory analyses of the MAIA scale with PCA, the result of the rotated component matrix for the Indian participants showed a different component/factor structure compared to the original English MAIA scale (Mehling et al., 2012) and the Spanish MAIA scale (Valenzuela Moguillansky and Reyes-Reyes, 2015), which had demonstrated equivalent factor structures. Our exploratory PCA showed an eight-component/factor model as the best model fit for the Indian participants. However, the low Cronbach's alpha value obtained on the eighth component, revealing a low internal consistency reliability of this component, prompted the exclusion of item 10 (one of the two items that made up the eighth component). The new PCA conducted, excluding only item 10 but including all the remaining 31 MAIA items, produced a structure of a seven-component/factor model as the best fit (Table 9). The cumulative variance explained by this seven-factor model in the Indian MAIA was 62.24%. The results of the rotated component matrix for the Spanish participants also showed a different component/factor structure compared to the original English and the Spanish version of the MAIA scale. These results demonstrated a seven-component/factor model with considerable differences in the grouping of the items compared with the original MAIA scale. In addition, the negative Cronbach's alpha value obtained in the seventh component prompted the exclusion of items 6 and 10 that made up this component. The results of the new PCA, excluding both items 6 and 10, indicated a good fit for a six-factor model (Table 10). The cumulative variance explained by this six-factor model in the Spanish MAIA was 69.02%.

**Table 9:** Exploratory PCA of the MAIA items in Indian participants.

	**FL**	**C**
**I**. **Attention Regulation** ***α*** **=** **0.867**		
11—I can pay attention to my breath without being distracted by things happening around me.	0.705	0.562
12—I can maintain awareness of my inner bodily sensations even when there is a lot going on around me.	0.568	0.513
13—When I am in conversation with someone, I can pay attention to my posture.	0.645	0.537
14—I can return awareness to my body if I am distracted.	0.732	0.653
15—I can refocus my attention from thinking to sensing my body.	0.754	0.684
16—I can maintain awareness of my whole body even when a part of me is in pain or discomfort.	0.702	0.641
17—I am able to consciously focus on my body as a whole.	0.595	0.630
**II**. **Emotional Awareness and Self-Regulation** ***α*** **=** **0.756**		
4—I notice changes in my breathing, such as whether it slows down or speeds up.	0.428	0.521
19—When something is wrong in my life I can feel it in my body.	0.444	0.486
20—I notice that my body feels different after a peaceful experience.	0.721	0.578
21—I notice that my breathing becomes free and easy when I feel comfortable.	0.780	0.665
22—I notice how my body changes when I feel happy / joyful.	0.776	0.692
23—When I feel overwhelmed I can find a calm place inside.	0.550	0.386
24—When I bring awareness to my body I feel a sense of calm.	0.578	0.521
**III**. **Body Listening and Self-Regulation** ***α*** **=** **0.833**		
18—I notice how my body changes when I am angry.	0.430	0.476
25—I can use my breath to reduce tension.	0.597	0.645
26—When I am caught up in thoughts, I can calm my mind by focusing on my body/breathing.	0.696	0.760
27—I listen for information from my body about my emotional state.	0.633	0.653
28—When I am upset, I take time to explore how my body feels.	0.742	0.601
29—I listen to my body to inform me about what to do.	0.566	0.652
**IV**. **Trusting** ***α*** **=** **0.836**		
30—I am at home in my body.	0.778	0.691
31—I feel my body is a safe place.	0.808	0.802
32—I trust my body sensations.	0.756	0.748
**V**. **Noticing_I** ***α*** **=** **0.715**		
1—I notice where in my body I am comfortable.	0.661	0.627
2—When I am tense I notice where the tension is located in my body.	0.813	0.725
3—I notice when I am uncomfortable in my body.	0.749	0.676
**VI**. **Not-Worrying_I** ***α*** **=** **0.639**		
8—When I feel physical pain, I become upset.	0.770	0.629
9—I start to worry that something is wrong if I feel any discomfort.	0.809	0.676
**VII**. **Not-Distracting** ***α*** **=** **0.611**		
5—I do not notice (I ignore) physical tension or discomfort until they become more severe.	0.753	0.602
6—I distract myself from sensations of discomfort.	0.769	0.659
7—When I feel pain or discomfort, I try to power through it.	0.689	0.602

**Table 10:** Exploratory PCA of the MAIA items in Spanish participants.

	**FL**	**C**
**I. Attention Regulation_S** **α** **=** **0.916**		
11—I can pay attention to my breath without being distracted by things happening around me.	0.768	0.721
12—I can maintain awareness of my inner bodily sensations even when there is a lot going on around me.	0.762	0.696
13—When I am in conversation with someone, I can pay attention to my posture.	0.737	0.636
14—I can return awareness to my body if I am distracted.	0.785	0.687
15—I can refocus my attention from thinking to sensing my body.	0.705	0.731
16—I can maintain awareness of my whole body even when a part of me is in pain or discomfort.	0.627	0.667
17—I am able to consciously focus on my body as a whole.	0.582	0.715
27—I listen for information from my body about my emotional state.	0.512	0.752
**II. Body Listening, Trusting and Self-Regulation** **α** **=** **0.933**		
3—I notice when I am uncomfortable in my body.	0.442	0.469
23—When I feel overwhelmed I can find a calm place inside.	0.645	0.600
24—When I bring awareness to my body I feel a sense of calm.	0.613	0.713
25—I can use my breath to reduce tension.	0.537	0.614
26—When I am caught up in thoughts, I can calm my mind by focusing on my body/breathing.	0.638	0.730
28—When I am upset, I take time to explore how my body feels.	0.584	0.780
29—I listen to my body to inform me about what to do.	0.588	0.805
30—I am at home in my body.	0.816	0.853
31—I feel my body is a safe place.	0.784	0.845
32—I trust my body sensations.	0.700	0.763
**III. Emotional Awareness_S** **α** **=** **0.852**		
4—I notice changes in my breathing, such as whether it slows down or speeds up.	0.373	0.370
18—I notice how my body changes when I am angry.	0.565	0.690
19—When something is wrong in my life, I can feel it in my body.	0.643	0.736
20—I notice that my body feels different after a peaceful experience.	0.757	0.702
21—I notice that my breathing becomes free and easy when I feel comfortable.	0.763	0.798
22—I notice how my body changes when I feel happy / joyful.	0.765	0.740
**IV. Noticing_S** **α** **=** **0.727**		
1—I notice where in my body I am comfortable.	0.808	0.739
2—When I am tense I notice where the tension is located in my body.	0.786	0.691
**V. Not_Worrying_S** **α** **=** **0.542**		
8—When I feel physical pain, I become upset.	0.777	0.646
9—I start to worry that something is wrong if I feel any discomfort.	0.675	0.546
**VI. Not_Distracting_S** **α** **=** **0.547**		
5—I do not notice (I ignore) physical tension or discomfort until they become more severe.	0.758	0.633
7—When I feel pain or discomfort, I try to power through it.	0.779	0.639

*FL, Factor loadings; C, Communalities; α = Cronbach's alpha*.

Low Cronbach's alphas for the Not-Distracting and Not-Worrying sub-scales, which include item 6 (I distract myself from sensations of discomfort) and item 10 (I can notice an unpleasant body sensation without worrying about it), respectively, have been reported in several studies of the original English version and its translations. This was in part explained by their two characteristics: (1) both scales have reversely scored or “negative” items and (2) both consist of only three items, and Cronbach's alpha is sensitive to the number of items. These subscales have been improved by the authors in a recent version of MAIA (Mehling et al., 2018). Nevertheless, the Cronbach's alpha values of the full MAIA scale obtained for the total sample (0.91), the Indian participants (0.89), and Spanish participants (0.93) were all adequate enough to confirm the internal consistency reliability of the scale in the total sample and in both the cultural samples.

The results of the exploratory PCA also showed a different factor composition in the MAIA scale for the Indian and the Spanish samples, which have been renamed in Tables 9, 10, based on the grouping of items. The factor composition being different in the two cultural samples led us to reject the examination of the underlying skills represented by the MAIA subscales when considering our first and second hypotheses.

## Discussion

The main objective of the study, as elaborated earlier, was to explain the self-regulatory mechanisms of action in yoga practice within the S-ART theoretical framework. Apart from this, the second and one of the empirical objectives of this study was to analyze the role of yoga practice on the self-regulatory mechanisms of action in yoga practitioners vs. NP through the study of psychological variables mapped to the three S-ART meta-abilities of *self-awareness, self-regulation, and self-transcendence*.

In relation to this, the results showed significantly better s*elf-awareness* and *self-regulatory* abilities in yoga practitioners (Indian and Spanish combined) than NP (as indicated by the scores on MAIA and EQ-D, respectively). Thus, the results obtained were in line with the previous findings, with a new contribution that this finding was obtained within a culturally diverse sample. Better *self-awareness* is reflected in a general higher level of interoceptive awareness in the yoga practitioners compared to NP. Previous research by Rani and Rao (1994) demonstrated an increase in awareness of the normal, non-emotive bodily process through the practice of *Hatha Yoga*. The previous studies, in which specific yoga programs were conducted with chronic pain patients, have also found increases in body awareness (Tul et al., 2011; Cramer et al., 2013).

In regard to *self-regulation*, we found a higher decentering ability in the yoga practitioners compared to NP. Decentering represents a metacognitive capacity, which includes three interrelated processes: meta-awareness, de-identification from internal experience, and reduced reactivity to thought content. It can be considered as an emotion regulation skill set, which develops in the process of *self-regulation*. The development of this skill set of decentering, arising through mindfulness-based interventions, is related to a decrease in subjective emotional reactions and amelioration of distress disorders (King and Fresco, 2019). A previous study by the first author of the current study, demonstrated improvement in decentering after yoga practice, along with significant improvements in interoceptive awareness (measured by MAIA), emotional symptomatology (anxiety, distress, depression symptoms, and perceived stress), and perception of happiness and satisfaction with life in a group of Spanish hypertensive patients, who followed a yoga intervention, compared with a control group (Tolbaños Roche et al., 2017).

In relation to the third dimension of *self-transcendence*, we did not find significant differences between yoga practitioners and NP, which was a deviation from the reviewed previous findings. This may have been due to different conceptualizations of *self-transcendence* in the previous studies compared to ours. We mapped the S-ART dimension of *self-transcendence* to multidimensional relational compassion, which included relationships of self-self, self-other, other-self, and other-other. This dimension of *self-transcendence*, which includes social components and possibly social outcomes of yoga practice such as prosocial behavior, needs more exploration, and related hypotheses need to be further elaborated in future studies (Gard et al., 2014).

Regarding our second objective, aimed at further studying how perseverance in yoga (long-term yoga practice) influenced these self-regulatory mechanisms, our results demonstrated that perseverance in yoga practice acted as a significant predictor of interoceptive awareness, a vital aspect of self-awareness, and self-regulatory abilities, in the yoga practitioners of the current study. Providing additional support to our results in this regard, the study of Villemure et al. (2013) showed that regular and long-term yoga practice improved pain tolerance in a North American sample by using cognitive strategies involving parasympathetic activation and interoceptive awareness to tolerate pain. In addition, a study with breast cancer survivors recruited from three comprehensive cancer care centers in Bengaluru, India, reported that participants with more than 6 months of regular yoga practice during the prior year of the intervention had better psychological profiles and were able to deal with demanding situations better than those who had attended <3 yoga sessions during the previous year (Amritanshu et al., 2017).

In relation to the third study objective of exploring the contribution of yoga practice in each cultural sample, the Indian and Spanish samples demonstrated interestingly divergent findings. The results showed that the practice of yoga and perseverance in it acted as a significant predictor of the abilities of interoceptive awareness and decentering in the Indian practitioners, who had more than 1 year of yoga practice, but not in Spanish practitioners. For the Spanish participants, physical exercise and frequency of yoga practice acted as better predictors of the abilities of interoceptive awareness (*self-awareness*) and decentering (*self-regulation*) than yoga practice and perseverance in it. Although our culturally diverse sample of yoga practitioners vs. NP was not as methodologically rigorous as it should have been, our findings do provide some form of reaffirmation to findings from cultural psychological research cited earlier on in the “Introduction” section, and signal to the need for future studies to take up culture as an important variable when conducting research with the culturally rooted practice of yoga. Furthermore, the different factor composition in the MAIA scale for the Indian and the Spanish samples showed in the exploratory PCA gives some evidence for cultural differences in psychological phenomena, which would be worth exploring in future research.

The obtained findings support the proposition made by the current researchers in the “Introduction” section to consider the S-ART model as a theoretical framework to explain the self-regulatory mechanisms of action in yoga practice. Yoga practitioners, in the current study, have shown a higher level of *self-awareness* and *self-regulation* compared to NP. The ability to be aware of one's own body's feelings and sensations and their relationships with emotions and thoughts may be the main mechanism in regulating unpleasant feelings, disturbances, and distress in the yoga practitioners. The yoga practitioners have also shown a higher decentering or non-attachment ability, one of the six neurocognitive mechanisms underlying the mindfulness process according to the S-ART model. The decentering attitude facilitates a greater willingness to experience negative emotions, that is, a greater tolerance of unwanted emotional states without deliberately trying to control them, consequently reducing emotional reactivity (Britton et al., 2012). Observing and accepting the emotions without trying to control emotional states should facilitate effective emotion regulation (Heppner et al., 2015). Thus, the leap forward from a control mode to one of acceptance, in which the present experience is accepted as a transitory experience, can be considered as a process of *self-regulation* in itself.

Although no significant differences between yoga practitioners and NP in *self-transcendence* (measured by the RCS) were found, the authors are of the opinion that this S-ART ability has more potential to emerge as a result of the uninterrupted and thoroughly committed practice of yoga, Therefore, *self-transcendence* could be probed further in future research with devoted long-term yoga practitioners.

Despite the novel application of the S-ART framework to yoga and the generation of testable hypotheses, the findings from the current study cannot be generalized due to non-random selection of targeted participants, non-equal selection of yoga practitioners and NP in the cross-cultural sample, and the use of self-reported measurements. Future research could use a more rigorous sampling method, and brain mapping and neurophysiological task-oriented measures when testing the hypotheses generated in the current study. It would be interesting if future studies could explore how the three S-ART meta-abilities in yoga practitioners with an advanced level of commitment not only just to the practices but also to following a completely yogic lifestyle.

## Conclusion

In the current study, applying the S-ART theoretical framework and model to yoga enabled the generation of testable hypotheses and further provided preliminary empirical evidence to explain the self-regulatory mechanisms of action in yoga practice in two contextually different cultures. Thus, the S-ART could potentially serve as an overarching theoretical framework in yoga similar to mindfulness research, and therefore, merits in-depth exploration in yoga research in the future.

## Data Availability Statement

The raw data supporting the conclusions of this article will be made available by the authors, without undue reservation.

## Ethics Statement

Ethical review and approval was not required for the study on human participants in accordance with the local legislation and institutional requirements. The patients/participants provided their written informed consent to participate in this study.

## Author Contributions

LT-R and PM contributed to the conception and design of the study, collected the data and wrote sections of the manuscript. PM organized the database. LT-R performed the statistical analysis and interpretation of data. LT-R wrote the first draft of the manuscript. Both authors contributed to manuscript revision, and read and approved the submitted version.

## Conflict of Interest

The authors declare that the research was conducted in the absence of any commercial or financial relationships that could be construed as a potential conflict of interest. The handling editor declared a shared affiliation, though no other collaboration, with one of the authors LT-R at the time of the review.

## Publisher's Note

All claims expressed in this article are solely those of the authors and do not necessarily represent those of their affiliated organizations, or those of the publisher, the editors and the reviewers. Any product that may be evaluated in this article, or claim that may be made by its manufacturer, is not guaranteed or endorsed by the publisher.
