# Corrigendum: Applying the S-ART Framework to Yoga: Exploring the Self-Regulatory Action of Yoga Practice in Two Culturally Diverse Samples

**DOI:** 10.3389/fpsyg.2021.778709

**Published:** 2021-11-26

**Authors:** Laura Tolbaños-Roche, Praseeda Menon

**Affiliations:** ^1^Department of Clinical Psychology, Psychobiology and Methodology, Section of Psychology, Faculty of Health Sciences, Universidad de La Laguna, San Cristóbal de La Laguna, Spain; ^2^Scientific Research Department, Kaivalyadhama Yoga Institute, Lonavala, India

**Keywords:** yoga, S-ART model, self-regulatory action, well-being, yoga practitioners

In the original article, there were a few mistakes regarding the tables. There was an error in the caption for **Table 4** as published. The original caption stated, “Bivariate correlations of the product variables…,” whereas it should be “Bivariate correlations of the outcome variables….” The corrected caption appears below.

**Table 4**. Bivariate correlations of the outcome variables with sociodemographic characteristics, life habits, and aspects of yoga practice in the Indian participants.

Similarly, in the original article, there was a mistake in the caption for **Table 5** as published. The original caption stated, “Bivariate correlations of the product variables…,” whereas it should be “Bivariate correlations of the outcome variables….” The corrected legend appears below.

**Table 5**. Bivariate correlations of the outcome variables with sociodemographic characteristics, life habits, and aspects of yoga practice in the Spanish participants.

Additionally, in the original article, there were mistakes in Table 9 as published. The third component BODY LISTENING AND SELF-REGULATION had not been numbered and the numbering of the components was wrong from TRUSTING onwards. The corrected Table 9 appears below.

**Table 9:** Exploratory PCA of the MAIA items in Indian participants.

	**FL**	**C**
**I**. **Attention Regulation** ***α*** **=** **0.867**		
11—I can pay attention to my breath without being distracted by things happening around me.	0.705	0.562
12—I can maintain awareness of my inner bodily sensations even when there is a lot going on around me.	0.568	0.513
13—When I am in conversation with someone, I can pay attention to my posture.	0.645	0.537
14—I can return awareness to my body if I am distracted.	0.732	0.653
15—I can refocus my attention from thinking to sensing my body.	0.754	0.684
16—I can maintain awareness of my whole body even when a part of me is in pain or discomfort.	0.702	0.641
17—I am able to consciously focus on my body as a whole.	0.595	0.630
**II**. **Emotional Awareness and Self-Regulation** ***α*** **=** **0.756**		
4—I notice changes in my breathing, such as whether it slows down or speeds up.	0.428	0.521
19—When something is wrong in my life I can feel it in my body.	0.444	0.486
20—I notice that my body feels different after a peaceful experience.	0.721	0.578
21—I notice that my breathing becomes free and easy when I feel comfortable.	0.780	0.665
22—I notice how my body changes when I feel happy / joyful.	0.776	0.692
23—When I feel overwhelmed I can find a calm place inside.	0.550	0.386
24—When I bring awareness to my body I feel a sense of calm.	0.578	0.521
**III**. **Body Listening and Self-Regulation** ***α*** **=** **0.833**		
18—I notice how my body changes when I am angry.	0.430	0.476
25—I can use my breath to reduce tension.	0.597	0.645
26—When I am caught up in thoughts, I can calm my mind by focusing on my body/breathing.	0.696	0.760
27—I listen for information from my body about my emotional state.	0.633	0.653
28—When I am upset, I take time to explore how my body feels.	0.742	0.601
29—I listen to my body to inform me about what to do.	0.566	0.652
**IV**. **Trusting** ***α*** **=** **0.836**		
30—I am at home in my body.	0.778	0.691
31—I feel my body is a safe place.	0.808	0.802
32—I trust my body sensations.	0.756	0.748
**V**. **Noticing_I** ***α*** **=** **0.715**		
1—I notice where in my body I am comfortable.	0.661	0.627
2—When I am tense I notice where the tension is located in my body.	0.813	0.725
3—I notice when I am uncomfortable in my body.	0.749	0.676
**VI**. **Not-Worrying_I** ***α*** **=** **0.639**		
8—When I feel physical pain, I become upset.	0.770	0.629
9—I start to worry that something is wrong if I feel any discomfort.	0.809	0.676
**VII**. **Not-Distracting** ***α*** **=** **0.611**		
5—I do not notice (I ignore) physical tension or discomfort until they become more severe.	0.753	0.602
6—I distract myself from sensations of discomfort.	0.769	0.659
7—When I feel pain or discomfort, I try to power through it.	0.689	0.602

In the original article, there were also several grammatical or typographical errors that could impact the meaning of the text. A number of corrections have been made, as outlined below.

A correction has been made to *Introduction, Mindfulness and Yoga, Paragraph 2*. The corrected paragraph is shown below.

The popularity of yoga, an Eastern (Indian) mind-body practice, has been growing year by year worldwide. Ways and means to regulate oneself effectively form the foundation of the path of yoga in the accomplishment of holistic health and well-being. However, in many cases, yoga, out of its cultural context, is used only for fitness or, at best, as a body-mind relaxing technique. Over the past two decades, a growing body of research proving yoga's healing benefits has promoted the development of yoga therapy as a clinical treatment for many health conditions (Pascoe and Bauer, 2015; Kumar et al., 2016; Haider et al., 2017; Park and Han, 2017; Thind et al., 2017; Falkenberg et al., 2018). Scientific evidence as well as the effort of many yoga institutions, teachers, and therapists, which have spread genuine yoga teaching for long, have contributed to the universal essence of yoga.

Corrections have been made to *Introduction, Culture as a Salient Contributor, Paragraphs 5, 6 and 7*. The corrected paragraphs are shown below.

Divergent self-construals in culturally diverse contexts, as mentioned earlier, are also likely to affect one's relationship with the self as well as that with others, an aspect that yoga also affects. When the relationships between self-compassion and relationally interdependent self-construal were examined, the interdependent self-construal was found to be positively related to self-kindness, common humanity, and mindfulness factors of self-compassion, and negatively correlated to self-judgment, isolation, and over-identification factors of self-compassion (Akin and Eroglu, 2013). In terms of social relatedness, a review of the evidence suggested that compared to European Americans, Asians and Asian Americans tend to shy away from explicit social support because of the concerns with potential negative relational consequences of such behavior (Kim et al., 2008).

Although the above studies make a strong case for culture as an influential variable in psychological studies, some studies have identified mindfulness as a cross-culturally similar psychological process. Ghorbani et al. (2009) found similar patterns of positive correlation of mindfulness with the self-reported adjustment comparing the participants from Iran (Middle Eastern culture) and the USA (Western culture). In yet another study, Ivtzan et al. (2018) found that after an 8-week online Mindfulness-Based Flourishing Program (MBFP), both British and Chinese (Hong Kong based) experimental groups showed significant improvements, with large effect sizes, in mindfulness, gratitude, self-compassion, meaning, and negative affect compared to a control group, providing preliminary evidence for the MBFP's cross-cultural validity. The authors concluded that the structured mindfulness-based programs lead to a similar cross-cultural pattern of benefits even when the initial understanding of mindfulness might have been different.

Despite the conflicting evidence related to culture-specific patterns in the field of mindfulness and yoga, cultural psychological research has “demonstrated that culture is implicated at a much more fundamental level of psychological processing than what was previously considered” (Heine and Norenzayan, 2006, p. 253). In fact, the inconsistent results in the above studies drive home the point that culture is an important factor to consider in psychological research, especially when culturally rooted practices, such as yoga, form a part of the research. Including culture as a factor in such psychological research will help accumulate a robust evidence-base, in turn determining the extent to which psychological processes related to mindfulness and yoga may be uniform across cultures or have culture-specific qualifiers, and thereby variations.

A correction has been made to *Introduction, The S-ART Model in Yoga, Paragraph 4 of Point 2*. The corrected paragraph is shown below.

An example of how therapy can be based on body-awareness is provided by the “model of sensorimotor therapy” (Ogden et al., 2009), wherein the integration of all levels of experience—sensorimotor, emotional, and cognitive—starts with an awareness of body sensations. In the same way, starting from a visceral, sensorial and motor awareness, and through the development of “bottom-up” as well as “top-down” awareness of emotional and cognitive processes, yoga practices act on all levels of experience-organization (physical, emotional and cognitive), facilitate their integration, and thereby bring about self-regulation and well-being. Supporting this idea is the previously cited study of Gard et al. (2014) regarding the potential self-regulatory mechanisms of yoga for psychological health. The authors posit that yoga practices would lead to self-regulation through both the promotion of behavioral changes as well as the inhibition of a non-desirable output by both higher-level and lower-level brain networks when facing stress-related challenges, with a consequent positive impact on health and well-being. In their review, the authors also provided sufficient empirical evidence for the association between yoga and self-regulation across cognitive, behavioral, autonomic, and emotion domains.

A correction has been made to *Introduction, Overview of the Present Study, Paragraphs 6 and 7*. The corrected paragraphs are shown below.

Previous evidence has shown positive results with yoga practice in shorter interventions. However, some of the studies have remarked the importance of regularity of practice, demonstrating that more practice was associated with lower mood disturbances (Khalsa et al., 2012), cognitive emotion regulation (Gootjes et al., 2011), and greater well-being and quality of sleep (Danhauer et al., 2009). Therefore, as a part of its second objective, our study aimed at studying how perseverance in yoga practice (long-term yoga practice) further contributed to the self-regulatory mechanisms of action in yoga.

There remains a substantive gap in our knowledge about how Western models of psychological processes, such as body consciousness, emerge—or fail to emerge—in other cultural contexts (Ma-Kellams, 2014). Moreover, we also found a lack of adequate studies looking at the role of different cultural contexts in yoga, particularly related to that of the self-regulatory action in yoga practice. Therefore, we thought that it is important to explore the role of yoga, within the S-ART model, in two culturally diverse contexts—that of the collectivistic Eastern culture of India and that of the individualistic Western culture of Spain. This formed part of our third exploratory objective, which could possibly help to generate worthwhile information for the formulation of hypotheses in future research.

Corrections have been made to *Introduction, Overview of the Present Study, Paragraph 9*. The corrected paragraph is shown below.

Each of the three abilities of the S-ART model was appropriately mapped to four psychological variables of interoceptive awareness (multidimensional), decentering, emotion regulation, and relational compassion, with the middle two variables used as indicators of self-regulation. These model-mapped variables were treated as our outcome psychological variables, and were measured by using the standard psychological instruments elaborated in the next section. Very few studies have attempted to take a multidimensional view of variables such as somatic awareness (one of the key skills implicated in interoceptive awareness) when dealing with different cultural contexts. Given the need for a greater precision in defining the nature of observed cultural variations, research would benefit with a more systematic use of multidimensional measures, for example, the Multidimensional Assessment of Interoceptive Awareness (MAIA; Mehling et al., 2012), in culturally diverse contexts (Ma-Kellams, 2014). In line with this suggestion, our study attempted to take a multidimensional view of the S-ART meta-abilities when selecting the psychological instruments to measure them.

Corrections have been made to *Methods, Data Analyses, Paragraphs 1, 2 and 3*. The corrected paragraphs are shown below.

The statistical package SPSS, version 15.0, was used for conducting the data analysis. A one-way ANOVA for the numerical variables and the chi-squared tests for the categorical variables were used for analyzing the homogeneity of the two groups of yoga practitioners and NP in sociodemographic characteristics of the total sample, life habits, and different aspects related to the practice of yoga. After this preliminary analysis, first, the Pearson correlation analysis was conducted to examine which of the measured sociodemographic characteristics, life habits, and aspects of yoga practice were significantly correlated with the four psychological outcome variables of Interoceptive Awareness, Decentering, Difficulties in Emotional Regulation (DERS), and Relational Compassion, as measured by MAIA, EQ-D, DERS, and RCS, respectively. Second, a one-way Multivariate ANOVA (MANOVA) was employed to test our first hypothesis and to determine whether there were differences between yoga practitioners and NP on the total scores of the outcome variables.

Next, we tested our second hypothesis related to the contribution of perseverance in yoga practice, measured by the length of yoga practice in months/years, on the psychological outcome variables, which showed significantly higher levels in yoga practitioners in the first hypothesis testing. The hierarchical regression analysis was conducted to explore the independent and additive power of perseverance in yoga practice when sociodemographic characteristics, life habits, and different aspects of yoga practice, which showed a significant correlation with these selected outcome variables, were controlled. In this regression analysis, the yoga practitioners were categorized into three groups: beginners (BG; between 1 month and <1 year of practice), medium-term practitioners (MP; between 1 and 5 years of practice), and long-term practitioners (LP; >5 years of practice).

Finally, we addressed our third objective of exploring the specific self-regulatory contribution of yoga in each cultural sample of Indian and Spanish participants. In order to fulfill this objective, first, the Pearson correlation analysis was conducted to examine which of the measured sociodemographic characteristics, life habits, and aspects of yoga practice in each of the Indian and Spanish samples were significantly associated with the four psychological outcome variables. Next, a one-way MANOVA was performed to determine whether there were differences between yoga practitioners and NP, on the total scores of the four outcome variables. In this MANOVA, the participants of each cultural sample were divided into three groups of NP (never or <1 month of yoga practice), BG (1 month to 1 year of yoga practice), and above 1- year practitioners (AYP; >1 year of yoga practice), so as to get further exploratory information on how yoga practice as well as perseverance in it would act on the outcome variables. As per the results obtained from this one-way MANOVA analysis on the total scores of the outcome variables, we conducted an exploratory principal components analysis (PCA) with Varimax rotation (extraction criterion: eigenvalue>1) only on the MAIA scale for each cultural sample. This exploratory PCA was done in order to examine whether the component/factor structure of the original MAIA version, represented by its subscales, would reproduce in both the Indian and Spanish samples, and in the event of divergence, to determine the MAIA component/factor structure of each cultural sample, enabling a more detailed examination of the culture-specific grouping of skills represented by its component subscales. Cronbach's alpha coefficient was used to assess the internal consistency reliability of the full MAIA scale in the total sample, as well as in the full MAIA scale and in its components (obtained from PCA) at each cultural sample. We also performed a hierarchical regression analysis in order to explore the independent and additive power of the practice of yoga and perseverance in it on the relevant outcome variables when sociodemographic characteristics, life habits, and different aspects of yoga practice, significantly correlated with these outcome variables, were controlled.

A correction has been made to *Results, Differences Among NP, BG, and AYP on the Total Scores of the Outcome Variables and the Results of the Hierarchical Regression Analysis in Each Cultural Sample, Paragraph 1*. The corrected paragraph is shown below.

The results of MANOVA showed a statistically significant multivariate effect on the total scores of MAIA, EQ-D, DERS, and RCS in the Indian participants (*F* = 2.530; *p* = 0.011; *df* = 8; power = 0.912). The tests of between-subjects effects displayed statistically significant differences among NP, BG, and AYP in the MAIA (*F* = 9.312; *p* < 0.001; *df* = 2; power = 0.976), and EQ-D scores (*F* = 6.384; *p* = 0.002; *df* = 2; power = 0.897). The results of the multiple comparisons (**Table 6**) showed significantly higher total scores on MAIA and EQ-D of only AYP in the Indian participants, indicating the influence of perseverance in yoga practice on the abilities of interoceptive awareness (*self-awareness*) and decentering (*self-regulation*) of Indian participants in the current study.

A correction has been made to *Discussion, Paragraph 5*. The corrected paragraph is shown below.

Regarding our second objective, aimed at further studying how perseverance in yoga (long-term yoga practice) influenced these self-regulatory mechanisms, our results demonstrated that perseverance in yoga practice acted as a significant predictor of interoceptive awareness, a vital aspect of self-awareness, and self-regulatory abilities, in the yoga practitioners of the current study. Providing additional support to our results in this regard, the study of Villemure et al. (2013) showed that regular and long-term yoga practice improved pain tolerance in a North American sample by using cognitive strategies involving parasympathetic activation and interoceptive awareness to tolerate pain. In addition, a study with breast cancer survivors recruited from three comprehensive cancer care centers in Bengaluru, India, reported that participants with more than 6 months of regular yoga practice during the prior year of the intervention had better psychological profiles and were able to deal with demanding situations better than those who had attended <3 yoga sessions during the previous year (Amritanshu et al., 2017).

A correction has been made to *Discussion, Paragraph 9*. The corrected paragraph is shown below.

Despite the novel application of the S-ART framework to yoga and the generation of testable hypotheses, the findings from the current study cannot be generalized due to non-random selection of targeted participants, non-equal selection of yoga practitioners and NP in the cross-cultural sample, and the use of self-reported measurements. Future research could use a more rigorous sampling method, and brain mapping and neurophysiological task-oriented measures when testing the hypotheses generated in the current study. It would be interesting if future studies could explore how the three S-ART meta-abilities in yoga practitioners with an advanced level of commitment not only just to the practices but also to following a completely yogic lifestyle.

In the original article, there were several errors involving the incorrect use of the word “product.” This should instead be “outcome.” A number of corrections have been made, as detailed below.

A correction has been made to the heading *Correlations of the Product Variables With Sociodemographic Characteristics, Life Habits, and Aspects of Yoga Practice* under *Results, Main Analyses*. The corrected heading is shown below.

“Correlations of the Outcome Variables With Sociodemographic Characteristics, Life Habits, and Aspects of Yoga Practice”

A correction has been made to *Results, Correlations of the Outcome Variables With Sociodemographic Characteristics, Life Habits, and Aspects of Yoga Practice, Paragraph 1*:

When the association of the four outcome variables with the sociodemographic characteristics, life habits, and aspects of yoga practice was analyzed, the results, shown in **Table 2**, indicated significant correlations of Interoceptive Awareness (MAIA) with marital status, physical exercise, frequency of practice, and type of (yoga) practices, that of decentering (EQ-D) with gender, education, weight, physical exercise, and frequency of practice, that of DERS with age, and physical exercise, and that of RCS with physical exercise.

A correction has also been made to the heading *Differences Between Yoga Practitioners and NP in the Product Variables Scores* under *Results, Main Analyses*. The corrected heading is shown below.

“Differences Between Yoga Practitioners and NP in the Outcome Variables Scores”

A correction has been made to *Results, Differences Between Yoga Practitioners and NP in the Outcome Variables Scores, Paragraph 1*. The corrected paragraph is shown below.

When testing our first hypothesis related to the differences between yoga practitioners and NP in the outcome variables, the results of MANOVA showed a significant multivariate effect on the total scores of the outcome variables of MAIA, EQ-D, DERS, and RCS (*F* = 6.305; *p* < 0.001; *df* = 4; power = 0.989). The tests of between-subject effects reported statistically significant differences between yoga practitioners and NP in the MAIA (*F* = 24.457; *p* < 0.001; *df* = 1; power = 0.999) and the EQ-D scores (*F* = 12.143; *p* = 0.001; *df* = 1; power = 0.935).

A correction has been made to the heading *Contribution of Perseverance in Practice of Yoga Practitioners on the Product Variables* under *Results, Main Analyses*. The corrected heading is shown below.

“Contribution of Perseverance in Practice of Yoga Practitioners on the Outcome Variables”

A correction has been made to the *heading Correlations of the Product Variables in Each Cultural Sample With Sociodemographic Characteristics, Life Habits, and Aspects of Yoga Practice*, under *Results, Additional Analyses: Exploration of the Contribution of Yoga Practice in Each Cultural Sample of Indian and Spanish Participants*. The corrected heading is shown below.

“Correlations of the Outcome Variables in Each Cultural Sample With Sociodemographic Characteristics, Life Habits, and Aspects of Yoga Practice”

A correction has been made to *Results, Correlations of the Outcome Variables in Each Cultural Sample With Sociodemographic Characteristics, Life Habits, and Aspects of Yoga Practice, Paragraph 1*. The corrected paragraph is shown below.

When the association of the four outcome variables with sociodemographic characteristics, life habits, and aspects of yoga practice were examined for each cultural sample (indicated by nationality), the results related to the Indian participants, shown in **Table 4**, indicated that Interoceptive Awareness (MAIA) was significantly correlated with smoking and physical exercise, and decentering (EQ-D) with height and physical exercise.

A correction has been made to the heading *Differences Among NP, BG, and AYP on the Total Scores of the Product Variables and the Results of the Hierarchical Regression Analysis in Each Cultural Sample*, under *Results, Additional Analyses: Exploration of the Contribution of Yoga Practice in Each Cultural Sample of Indian and Spanish Participants*. The corrected heading is shown below.

“Differences Among NP, BG, and AYP on the Total Scores of the Outcome Variables and the Results of the Hierarchical Regression Analysis in Each Cultural Sample”

A correction has also been made to *Results, Exploratory Factor Analysis of the MAIA Scale in Each Cultural Sample, Paragraph 1*. The corrected paragraph is shown below.

It was important to understand the similarity or difference of factor structure of the MAIA scale in each cultural sample as it emerged as a significant outcome variable in the aforementioned MANOVA analysis in both the Indian and Spanish samples.

Additionally, there was a referencing error in the original article. The citation and reference listed for Menezes et al. required amendment, as detailed below.

In the original article, two references, 2015a and 2015b, were included for Menezes et al. Only the 2015a reference is required. Therefore, the following corrections have been made.

The original reference, “Menezes, C. B., Dalpiaz, N. R., Kiesow, L. G., Sperb, W., Hertzberg, J., and Oliveira, A. A. (2015a). Yoga and emotion regulation: a review of primary psychological outcomes and their physiological correlates. Psychol. Neurosci. 8, 82–101. 10.1037/h0100353” has been amended to be “Menezes, C. B., Dalpiaz, N. R., Kiesow, L. G., Sperb, W., Hertzberg, J., and Oliveira, A. A. (2015). Yoga and emotion regulation: a review of primary psychological outcomes and their physiological correlates. Psychol. Neurosci. 8, 82–101. 10.1037/h0100353.”

The reference “Menezes, C. B., Dalpiaz, N. R., Rossi, N. T., and De Oliveira, A. A. (2015b). Yoga and the interplay between attentional load and emotion interference. Psychol. Rep. 117, 271–289. 10.2466/28.02.PR0.117c16z1” has been deleted from the article.

A correction has been made to *Introduction, Overview of the Present Study, Paragraph 4*. The corrected paragraph is shown below.

Regarding evidence related to the second S-ART meta-ability of self-regulation, in a review of 24 articles on the emotion regulation potential of yoga practice, Menezes et al. (2015) found evidence of the effect of yoga in the improvement of emotional functioning in both healthy subjects and people who suffer from different health conditions. This evidence suggests that yoga can help to promote healthier psychological responses and it is a potential emotion regulation strategy working through mechanisms such as reappraisal, attention regulation, self-monitoring, self-awareness, and autonomic regulation. Gard et al. (2014), based on evidence reviewed from various studies, in which yoga aided and promoted self-regulation across cognitive, emotional, behavioral, and autonomic domains, proposed a model for the self-regulatory mechanisms of yoga in well-being. Previous research by the first author of the current study also explored the role of yoga on self-regulation, showing that practicing yoga had a beneficial effect on physiological, emotional, and cognitive self-regulation in patients who suffered from essential arterial hypertension (Tolbaños Roche et al., 2017).

The authors state that these errors do not change the scientific conclusions in any way. The original article has been updated.

## Publisher's Note

All claims expressed in this article are solely those of the authors and do not necessarily represent those of their affiliated organizations, or those of the publisher, the editors and the reviewers. Any product that may be evaluated in this article, or claim that may be made by its manufacturer, is not guaranteed or endorsed by the publisher.

## References

[B1] Akin A. Eroglu Y. (2013). Self-compassion and relational-interdependent self-construal. Stud. Psychol. 55:111. 10.21909/sp.2013.02.62934282977

[B2] Amritanshu R. R. Rao R. M. Nagaratna R. Veldore V. H. Usha Rani M. U. Gopinath K. S.. (2017). Effect of long-term yoga practice on psychological outcomes in breast cancer survivors. Indian J Palliative Care 23, 231–236. 10.4103/IJPC.IJPC_93_17 28827924 PMC5545946

[B3] Danhauer S. C. Mihalko S. L. Russell G. B. Campbell C. R. Felder L. Daley K.. (2009). Restorative yoga for women with breast cancer: findings from a randomized pilot study. Psycho-Oncol. 18, 360–368. 10.1002/pon.1503 19242916 PMC3930083

[B4] Falkenberg R. I. Eising C. Peters M. L. (2018). Yoga and immune system functioning: a systematic review of randomized controlled trials. J. Behav. Med. 41, 467–482. 10.1007/s10865-018-9914-y 29429046

[B5] Gard T. Noggle J. J. Park C. L. Vago D. R. Wilson A. (2014). Potential self-regulatory mechanisms of yoga for psychological health. Front. Hum. Neurosci. 8:770. 10.3389/fnhum.2014.00770 25368562 PMC4179745

[B6] Ghorbani N. Watson P. J. Weathington B. L. (2009). Mindfulness in Iran and the United States: cross-cultural structural complexity and parallel relationships with psychological adjustment. Curr. Psychol. 28:211. 10.1007/s12144-009-9060-3

[B7] Gootjes L. Franken I. H. A. Van Strien J. W. (2011). Cognitive emotion regulation in yogic meditative practitioners: sustained modulation of electrical brain potentials. J. Psychophysiol. 25, 87–94. 10.1027/0269-8803/a000043

[B8] Haider T. Sharma M. Branscum P. (2017). Yoga as an alternative and complementary therapy for cardiovascular disease: a systematic review. J. Evid. Based Complementary Altern. Med. 22, 310–316. 10.1177/2156587215627390 26787730 PMC5871178

[B9] Heine S. J. Norenzayan A. (2006). Toward a psychological science for a cultural species. Perspect. Psychol. Sci. 1, 251–269. 10.1111/j.1745-6916.2006.00015.x 26151632

[B10] Ivtzan I. Young T. Lee H. C. Lomas T. Daukantaite D. Kjell O. N. (2018). Mindfulness based flourishing program: a cross-cultural study of Hong Kong Chinese and British participants. J. Happiness Stud. 19, 2205–2223. 10.1007/s10902-017-9919-1

[B11] Khalsa S. B. Hickey-Schultz L. Cohen D. Steiner N. Cope S. (2012). Evaluation of the mental health benefits of yoga in a secondary school: a preliminary randomized controlled trial. J. Behav. Health. Ser. R. 39, 80–90. 10.1007/s11414-011-9249-8 21647811

[B12] Kim H. S. Sherman D. K. Taylor S. E. (2008). Culture and social support. Am. Psychol. 63:518. 10.1037/0003-066X 18793039

[B13] Kumar V. Jagannathan A. Philip M. Thulasi A. Angadi P. Raghuram N. (2016). Role of yoga for patients with type II diabetes mellitus: a systematic review and meta-analysis. Complement. Ther. Med. 25, 104–112. 10.1016/j.ctim.2016.02.001 27062957

[B14] Ma-Kellams C. (2014). Cross-cultural differences in somatic awareness and interoceptive accuracy: a review of the literature and directions for future research. Front. Psychol. 5:1379. 10.3389/fpsyg.2014.01379 25520688 PMC4253951

[B15] Mehling W. E. Price C. Daubenmier J. J. Acree M. Bartmess E. Stewart A. (2012). The multidimensional assessment of interoceptive awareness (MAIA). PLoS ONE 7:e.48230. 10.1371/journal.pone.0048230 23133619 PMC3486814

[B16] Menezes C. B. Dalpiaz N. R. Kiesow L. G. Sperb W. Hertzberg J. Oliveira A. A. (2015). Yoga and emotion regulation: a review of primary psychological outcomes and their physiological correlates. Psychol. Neurosci. 8, 82–101. 10.1037/h0100353

[B17] Ogden P. Minton K. y Pain C. (2009). El trauma y el cuerpo: Un modelo sensoriomotriz de psicoterapia. Bilbao: Desclée de Brouwer.

[B18] Park S. H. Han K. S. (2017). Blood pressure response to meditation and yoga: a systematic review and meta-analysis. J. Altern. Complem. Med. 23, 685–695. 10.1089/acm.2016.0234 28384004

[B19] Pascoe M. C. Bauer I. E. (2015). A systematic review of randomised control trials on the effects of yoga on stress measures and mood. J. Psychiat. Res. 68, 270–282. 10.1016/j.jpsychires.2015.07.013 26228429

[B20] Thind H. Lantini R. Balletto B. L. Donahue M. L. Salmoirago-Blotcher E. Bock B. C.. (2017). The effects of yoga among adults with type 2 diabetes: a systematic review and meta-analysis. Prev. Med. 105, 116–126. 10.1016/j.ypmed.2017.08.017 28882745 PMC5653446

[B21] Tolbaños Roche L. Miró Barrachina M. T. Ibáñez Fernández I. Betancort M. (2017). YOGA and self-regulation in management of essential arterial hypertension and associated emotional symptomatology: a randomized controlled trial. Complement. Ther. Clin. Pract. 29, 153–161. 10.1016/j.ctcp.2017.09.012 29122254

[B22] Villemure C. Ceko M. Cotton V. A. Bushnell M. C. (2013). Insular cortex mediates increased pain tolerance in yoga practitioners. Cereb. Cortex 24, 2732–2740. 10.1093/cercor/bht124 23696275 PMC4153807

